# Comp34 displays potent preclinical antitumor efficacy in triple-negative breast cancer via inhibition of NUDT3-AS4, a novel oncogenic long noncoding RNA

**DOI:** 10.1038/s41419-020-03235-w

**Published:** 2020-12-11

**Authors:** Qiongyu Hao, Piwen Wang, Pranabananda Dutta, Seyung Chung, Qun Li, Kun Wang, Jieqing Li, Wei Cao, Wenhong Deng, Qing Geng, Katrina Schrode, Magda Shaheen, Ke Wu, Donghui Zhu, Qiao-Hong Chen, Guanglin Chen, Yahya Elshimali, Jay Vadgama, Yong Wu

**Affiliations:** 1 Division of Cancer Research and Training, Department of Internal Medicine, Charles Drew University of Medicine and Science, David Geffen UCLA School of Medicine and UCLA Jonsson Comprehensive Cancer Center, Los Angeles, CA 90095 USA; 2 Department of Oncology, Shanghai East Hospital, Tongji University School of Medicine, 200120 Shanghai, China; 3 Department of Breast Cancer, Cancer Center, Guangdong Provincial People’s Hospital & Guangdong Academy of Medical Sciences, 510080 Guangzhou, China; 4 Department of General Surgery, Renmin Hospital of Wuhan University, 430060 Wuhan, China; 5 Department of Thoracic Surgery, Renmin Hospital of Wuhan University, 430060 Wuhan, China; 6 Department of Biomedical Engineering, Stony Brook University, Stony Brook, NY 11794 USA; 7 Department of Chemistry, California State University, Fresno, 2555 E. San Ramon Avenue, M/S SB70, Fresno, CA 93740 USA

**Keywords:** Drug discovery, Diseases

## Abstract

The abnormal PI3K/AKT/mTOR pathway is one of the most common genomic abnormalities in breast cancers including triple-negative breast cancer (TNBC), and pharmacologic inhibition of these aberrations has shown activity in TNBC patients. Here, we designed and identified a small-molecule Comp34 that suppresses both AKT and mTOR protein expression and exhibits robust cytotoxicity towards TNBC cells but not nontumorigenic normal breast epithelial cells. Mechanically, long noncoding RNA (lncRNA) AL354740.1-204 (also named as NUDT3-AS4) acts as a microRNA sponge to compete with AKT1/mTOR mRNAs for binding to miR-99s, leading to decrease in degradation of AKT1/mTOR mRNAs and subsequent increase in AKT1/mTOR protein expression. Inhibition of lncRNA-NUDT3-AS4 and suppression of the NUDT3-AS4/miR-99s association contribute to Comp34-affected biologic pathways. In addition, Comp34 alone is effective in cells with secondary resistance to rapamycin, the best-known inhibitor of mTOR, and displays a greater in vivo antitumor efficacy and lower toxicity than rapamycin in TNBC xenografted models. In conclusion, NUDT3-AS4 may play a proproliferative role in TNBC and be considered a relevant therapeutic target, and Comp34 presents promising activity as a single agent to inhibit TNBC through regulation of NUDT3-AS4 and miR-99s.

## Introduction

Triple-negative breast cancers (TNBCs) account for 20% of all breast cancers (BCs) and are associated with adverse clinical outcomes^1^. Due to little therapeutic progress over the past decades, chemotherapy is still the standard of care for TNBC^2^. The abnormal AKT/mammalian target of rapamycin (AKT/mTOR) pathway is one of the most common genomic abnormalities in BCs including TNBC^3^, contributing to cancer progression and resistance to existing treatments^4,5^. Hence, targeting the AKT/mTOR pathway was considered to be an effective strategy for the treatment of TNBC recently.

Competing Endogenous RNAs (ceRNAs), including mRNAs, long noncoding RNAs (lncRNA)^6^, circular RNA (circRNA)^7^, regulate other RNA transcripts by competing for a pool of shared microRNAs^8^. ceRNA acts as a molecular sponge of microRNAs through its miRNA response elements (MRE), thus inhibiting the expression of miRNA target genes. Recent studies on solid tumors and hematopoietic malignancies reveal that ceRNAs plays an important role in the pathogenesis of tumors through changing the expression of crucial oncogenes or tumor suppressor genes^9,10^. Identification of the characteristics, functions and mechanisms of ceRNAs will not only further deepen our basic understanding of the pathogenesis of RNA-mediated cancer, but may also provide clues for the development of novel RNA-based therapeutic strategies for cancer.

The miR-99 family has three members (miR-99a, miR-99b, and miR-100) located in different chromosomal regions of human genome. miR-99a/b are commonly lost or inhibited in various human cancers^11,12^. miR-100 has been found to be a diagnostic and/or prognostic marker for human cancer due to its dysregulation and aberrant expression in many types of cancer^13–16^. Several studies have linked the miR-99 family to the regulation of AKT/mTOR signaling pathway^17–21^. Although previous studies have shown that deregulation of miR-99 family members may play key roles in various cancer types, these studies often focus on the downstream pathway of miR-99s and may only reflect a fraction of the biological attributes of this microRNA family. Consequently, many functional miR-99 family target genes still need to be determined, and the roles of upstream regulators of miR-99s have not been elucidated at all.

Here, we designed and synthesized a series of curcumin analogs and identified (1*E*,4*E*)−1,5-bis(1-(pentan-2-yl)-1*H*-imidazol-2-yl)penta-1,4-dien-3-one (Comp34) as a potent antitumor compound with a robust activity of inhibiting AKT1/mTOR mRNA and protein expression in preclinical models of TNBC. Mechanically, lncRNA-NUDT3-AS4 acts as a microRNA sponge to compete miR-99s with *AKT1/mTOR* mRNAs, leading to decrease in degradation of *AKT1/mTOR* mRNAs and subsequent increase in AKT1/mTOR protein expression. Comp34, via inducing endogenous NUDT3-AS4 reduction and miR-99s disassociation from NUDT3-AS4, promotes miR-99s-dependent degradation of *AKT1/mTOR* mRNA, subsequently inhibiting AKT1/mTOR protein expression and TNBC progression.

## Materials and methods

### Cell culture

The breast cancer cell lines MDA-MB-231, MDA-MB-468, T47D, MCF-7 and the human mammary epithelial cell line MCF-12A, and nontumorigenic (fibrocystic disease) mammary gland epithelial cell line MCF-10A were purchased from the ATCC (Rockville, MD). These cells were authenticated by Laragen, Inc. (Culver City, CA), by short tandem repeat (STR) profiling and monitoring cell morphology and biological behavior, and tested to exclude mycoplasma contamination before experiments. MDA-MB-231, MDA-MB-468, T47D, MCF-7 cells were cultured in DMEM high glucose (Cat: 11965092, Gibco™) supplemented with 10% FBS (Cat: 16000044, Gibco™) and 1% penicillin/streptomycin (Cat:15140122, Gibco™). MCF-12A cells were cultured in DMEM/F12 medium (Lonza) supplemented with 100 ng/mL cholera toxin. MCF-10A were cultured in MEBM medium (Lonza) supplemented with 100 ng/mL cholera toxin. T47D cells were maintained in RPMI1640 medium (Lonza), 10% FBS, and 1% penicillin/streptomycin.

### Establishment of the Rapamycin-resistant MDA-MB-231 cell lines

Rapamycin-resistant cell line was established from its parental cell line MDA-MB-231. MDA-MB-231 cells were exposed to a stepwise increasing concentration of rapamycin (Cat: tlrl-rap, InvivoGen). The medium with different concentration of chemicals was exchanged every 2 days, and the cells were cultured at least three generations in each step. The resistant cell lines were continuously cultured for at least 6 months until resistant colonies emerged.

### Human phospho-kinase array assay

Human Phospho-Kinase Array Kit (Cat: ARY003B, R&D Systems) was used to determine the relative levels of protein phosphorylation according to the manufacturer’s instructions. Forty-three kinase phosphorylation sites and two related total proteins were carefully selected by the Phospho-Kinase Array Kit using cell lysates prepared from cell lines known to express the target proteins.

### Vectors construction

The cDNA encoding NUDT3-AS4 was PCR-amplified from human blood genomic DNA and sub-cloned into the Hind III site of pcDNA3.1 (−) + SLNCR1 + 12x MS2 bs (Cat: 86828, addgene), named pcDNA3.1- NUDT3-AS4-MS2. The pcDNA3.1- NUDT3-AS4-MS2 with mutations in the miR-99s response elements was generated using Site Directed Mutagenesis by PCR and named pcDNA3.1- NUDT3-AS4-Mut1&2-MS2 (miR-99s). pcDNA3.1 (−) + SLNCR1 + 12x MS2 bs was digested with Hind III, then self-connected to generate pcDNA3.1-MS2. The full length region of either NUDT3-AS4 was amplified using PCR and sub-cloned into the psiCHECK™-2 vector (Promega, Madison, WI) for Luciferase reporter assay. The psiCHECK™-2_ NUDT3-AS4 with mutations in the miR-99s response elements was generated using Site Directed Mutagenesis by PCR and named psiCHECK™-2_ or NUDT3-AS4-Mut1&2-MS2 (miR-99s). The 3’ untranslated regions (3’-UTR) of *AKT1* mRNA containing the intact miR-99s recognition sequences were PCR-amplified and sub-cloned into psiCHECK™-2 (Promega, Madison, WI). The psiCHECK™-2_ *AKT1*_3’-UTR with mutations in the miR-99s response elements was generated using Site Directed Mutagenesis by PCR and named psiCHECK™-2_ *AKT1*_3’-UTR_Mut (miR-99s).

### Construction and transduction of lentiviral shRNAs

Three pairs of cDNA oligonucleotides to suppress NUDT3-AS4 expression were designed and synthesized, and the sequences of shRNA were shown in Table S2. The design of shRNAs was assisted by using web-based software provided by Invitrogen (http://rnaidesigner.invitrogen.com/rnaiexpress/). The shRNAs were constructed using pLKO.1 vector between the EcoR1 and Age1 cutting site according to Addgene TRC Cloning Protocol. After annealing, double-strand oligo was inserted into the liner lentiviral vector pLKO.1 vector. To produce the lentivirus suppressing NUDT3-AS4, HEK-293FT cells were cotransfected with the resulting vector, pGag/Pol, pRev, and pVSV-G using Lipofectamine 2000 according to the manufacturer’s guidelines. Infectious lentiviruses were harvested at 48 h post transfection and filtered through 0.45 µm PVDF filters and then designated as NUDT3-AS4_sh1, NUDT3-AS4_sh2, NUDT3-AS4_sh3. A scrambled shRNA was used as the negative control and designated as shRNA-control. Recombinant lentiviruses were concentrated 100-fold by ultracentrifugation (2 h at 50,000 × *g*). The virus-containing pellet was dissolved in DMEM, aliquoted and stored at −80 °C. MDA-MB-231 cells were infected with NUDT3-AS4_sh1, NUDT3-AS4_sh2, NUDT3-AS4_sh3 or shRNA-control in the presence of 8 μg/ml polybrene (Sigma-Aldrich). The supernatant was replaced with complete culture media after 48 h. After infection, puromycin (1.5 μg/ml) was used to select stably transduced cells. The expression of NUDT3-AS4 in the infected cells was confirmed by qRT-PCR 96 h after infection.

### Transient transfection

Transfections were performed using the Lipofectamine 3000 kit (Invitrogen) according to the manufacturer’s instructions. The double-stranded microRNA mimics and their respective negative control RNAs (Cat: HMI0023; HMI0983; HMI0985; HMC0002, SIGMA-ALDRICH INC) were introduced into cells at a final concentration of 50 nM.

### Western blot analysis

Cells were lysed in RIPA buffer (Cat#: 9806, CST) supplemented with protease and phosphatase inhibitor cocktail (Cat#: 78441B, Halt™ Thermo Scientific) and proteins (40 μg) were separated on 10% SDS/PAGE gel and then transferred onto PVDF membranes (Cat: LC2002, Thermo Scientific). After blocking with 5% non-fat milk, membranes were incubated at 4 °C overnight with appropriate dilutions of specific primary antibodies: mTOR (Cat#: 2972, CST), AKT1 (Cat#: 9272, CST), p-p70S6K (T389) (Cat#: 9205, CST), p-S6 (S235/236) (Cat#: 4858, CST), p-4EBP1 (S65) (Cat#: 9451, CST), HSP27 (Cat #: MA3-015, Invitrogen), BASP1 (Cat #: PA5-26969, Invitrogen), PHB2 (Cat#: 12295-1-AP, Proteintech), CCT6A (Cat#: ab140142, Abcam), HSPA8 (Cat#: clone N69, Sigma-Aldrich), SND1 (Cat#: 10760-1-AP, Proteintech), and β-actin (Cat#: 4970, CST) as loading control. After washing, the blots were incubated with HRP-conjugated secondary antibodies (Cat#: G-21040, Cat#: G-21234, invitrogen) and then visualized using the ECL system (Cat#: 32106, Pierce) on LI-COR system. Each blot shows a representative image that was obtained from at least three independent experiments.

### RNA extraction, reverse transcription, and quantitative real-time PCR analysis

For RNA extraction, total RNAs were isolated using RNeasy Mini Kit (Cat: 74104, QIAGEN), and microRNAs were isolated using the miRNeasy Mini Kit (Cat: 217004, QIAGEN) according to the manufacturer’s instructions. For Reverse transcription, the cDNA was synthesized from 1 µg of total RNA using iScript™ Reverse Transcription Supermix (Cat: 1708840, Bio-Rad). Quantitative real-time PCR (qRT-PCR) was performed using Universal SYBR^®^ Green Supermix (Cat: 1725270 Bio-Rad) on the CFX96 Real-Time PCR Detection Systems (Bio-Rad). TaqMan™ Advanced miRNA cDNA Synthesis (Cat: A28007, Applied Biosystems™) and TaqMan Advanced miRNA Assays (Cat: Assay ID 478519_mir; Assay ID 478343_mir; Assay ID 478224_mir; Assay ID 478594_mir, Applied Biosystems™) were used for microRNA expression analysis. Expression levels were normalized to the expression of 18 s (mRNA) or miR-320 (miRNA). The primers used in this study were summarized in the Table S3.

### Mammosphere formation assays

Single cells were plated in six-well ultra-low attachment plates (Corning Incorporated, Corning, NY, USA) at a density of 20,000 viable cells/ml. Cells were grown in StemFlex™ medium (Cat: A3349401, Gibco™). Pictures were taken to assess the ratio of spheres to aggregates of cells. Mammospheres were collected by gentle centrifugation (800 r.p.m.) after 10 days, and were fractionated by size using 100 mm cell strainers (BD Pharmingen™) and counted microscopically. To quantify proliferation, cells were seeded on day 0 at 50,000 cells/well in a six-well dish in duplicate. Cells were fixed and stained with 0.1% crystal violet and quantified by extraction in 10% acetic acid and spectrophotometer reading at optical density 600 nm.

### Flow cytometric assays

**Fig. 1:**
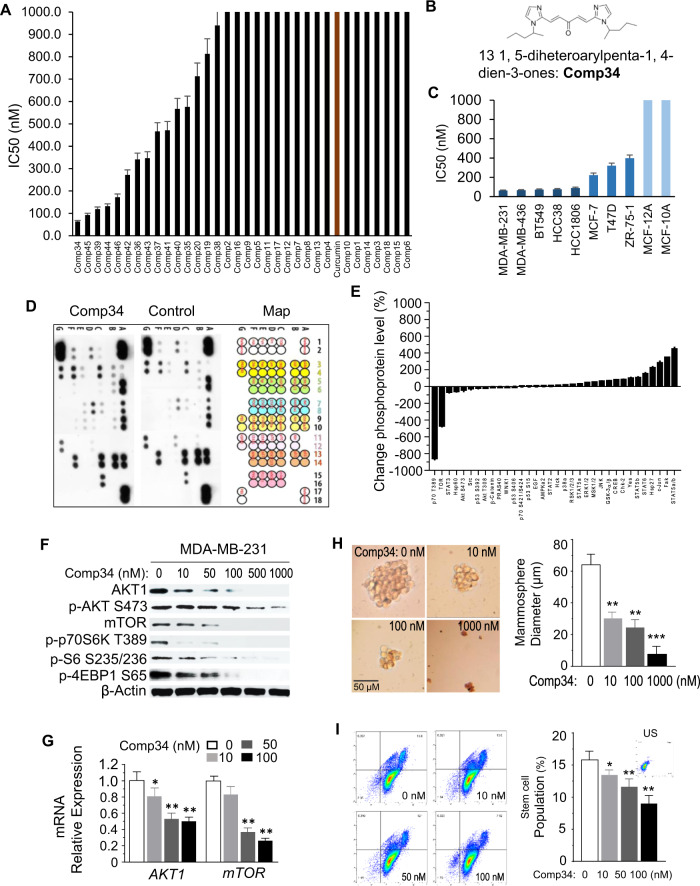
Comp34 inhibits TNBC cell growth, AKT/mTOR signaling pathway, and stemness. **a** Screening growth-inhibitory effects of 33 curcumin derivatives on MDA-MB-231 cells. **b** Chemical structure of Comp34. **c** IC_50_ values exhibited by Comp34 for cytotoxicity against various breast cancer cell lines and nontumorigenic breast epithelial cells. **d** Phosphorylation status of 34 kinases or transcription factors was assessed by the proteome profiler human phospho-kinase array. **e** Relative phosphorylation of spots was quantified by normalizing pixel density of the positive control to 100. **f** Western blotting (WB) analysis of the expression of AKT1, p-AKT, mTOR, p-p70S6K, p-S6, p-4EBP1 in the MDA-MB-231 cell line upon different concentrations of Comp34 treatment. **g** Reduction of *AKT1* and *mTOR* mRNA expression in MDA-MB-231 cells treated with Comp34 analyzed by qRT-PCR (*n* = 3 replicates). *P < 0.05 versus control. **h** Dissociation of mammospheres after treatment with Comp34. The results represent the mean diameter of mammospheres ± SD, *n* = 6. **i** Comp34 reduced the stemness of MDA-MB-231 cells in a dose-dependent manner. Stem cell populations were analyzed by FACS using CD44 and CD24 antibodies. US unstained. The results represent the mean percentage of stem cell population ± SD, *n* = 6.

Nonconfluent cultures were trypsinized into single-cell suspension, counted, washed with phosphate-buffered saline (PBS) supplemented with 5% FBS, and stained with antibodies specific for human cell surface markers. Briefly, a total of 250,000 cells were incubated with antibodies for 25 min at room temperature. Antibodies specific to human cell surface markers: phycoerythrin (PE)-conjugated antihuman CD24 mAb (Cat: 555428, BD Pharmingen™) and Fluorescein isothiocyanate (FITC)-conjugated antihuman CD44 mAb (Cat: 555478, BD Pharmingen™) were used to identify breast cancer cell surface antigen markers. The gating strategy for isolating CD44^+^CD24^low+^ was as shown in Fig. 1i. Unbound antibody was washed off and cells were processed for flow cytometry analysis no longer than 1 h post staining on an Attune™ NxT Flow Cytometer (Thermo Fisher Scientific), and data were analyzed with FlowJo software.

### Luciferase reporter gene assay

MDA-MB-231 cells were plated in 24-well plates and the plasmid was transfected into cells using Lipofectamine 3000 (Cat: L3000001, Invitrogen) according to the manufacturer’s instructions. psiCHECK-2 was used as internal control. NUDT3-AS4 was cloned into the psiCHECK-2 vector construct (Promega) downstream of the *Renilla* luciferase ORF. After 24 h, cells were lysed with lysis buffer and measured using the Dual-Luciferase Reporter Assay System (Promega) according to the manufacturer’s instructions. Site-directed mutation of NUDT3-AS4 binding site was designed and generated by PCR. pRL-CMV_*mTOR*_3’-UTR and pRL-CMV_*mTOR*_3’-UTR_Mut (miR-99s) were gifts from Dr. Dutta^21^.

### RNA FISH

20-nt long single-stranded oligo probes labeled with a particular fluorophore can hybridize to the complementary sequences in the lncRNA, so that the fluorescence signal can then be detected by fluorescence microscopy. The probe sequences were shown in table S3c. Briefly, MDA-MB-231 cells were fixed in 4% paraformaldehyde on a coverglass at room temperature for 15 min, then made permeable after rinsing with DEPC-PBS, in TritonX-100/DEPC/PBS (0.5%) for 5 min. Probes were diluted and applied at 200 nM in hybridization buffer (4xSSC, 0.5 mM EDTA, 10% dextran sulfate, 10–25% deionized-formamide in DEPC-H_2_O) and incubated at room temperature for 10 min or overnight at 4 °C. After hybridization, cells were washed in 2XSSC, 3 × 15 min and PBS, 3 × 15 min at room temperature. The hybridized samples were mounted onto glass slides with ProLong self-solidifying mounting medium. The samples finally were imaged using a fluorescent microscope to localize and detect RNA in situ.

### RNA immunoprecipitation (RIP)

MDA-MB-231 cells were cotransfected with pcDNA3.1-MS2, pcDNA3.1-MS2- NUDT3-AS4, pcDNA3.1-MS2- NUDT3-AS4 –Mut (miR-99s) and pMS2-GST. After 36 h, cells were used to perform RNA immunoprecipitation (RIP) experiments and RNA purification using GSH agarose beads (GE Healthcare) and the Magna RIP™ RNA-Binding Protein Immunoprecipitation Kit (Millipore, Bedford, MA) as described previously^22^. pMS2-GST and pMS2 plasmid were gifts from Dr. Gorospe^22^. The RNA fraction isolated by RIP was quantified by NanoDrop ND1000 (Thermo-Fisher Scientific, Waltham, MA) and its quality was assessed by Agilent 2100 Bioanalyzer (Agilent).

### Proliferation assay

Cell proliferation was determined by the CellTiter MTS assay (Promega) or Label-Free Cell Counting Kinetic Proliferation Assay.

CellTiter MTS assay (Promega): Cells were seeded in 96-well plates at a density of 5000 cells per well before performing the MTS assay according to the manufacturer’s instructions.

Label-Free Cell Counting Kinetic Proliferation Assay: Cells were seeded in 96-well plates at a density of 5000 cells. Cell culture plates were transferred by the BioSpa 8 to a Cytation™ 5 Cell Imaging Multi-Mode Reader (BioTek Instruments, Winooski, VT) every 4 h. Environmental conditions were maintained at 37 °C and 5% CO_2_ within the Cytation 5 throughout the imaging steps. Two high contrast brightfield images were captured at each time point: an in focus image used for reference, and a defocused image for cell counting. Label-free methods of measuring cell growth kinetics are preferable over the use of stains that can influence proliferation rates. Although confluence level can be used for some applications, cell counting is the most direct quantitative measure of cell proliferation over a broad range of cell population densities

### Synthesis and characterization of PLGA-Comp34 NPs

PLGA-Comp34 NPs were synthesized by standard single emulsion techniques as described before^23^. Briefly, a 10% w/w drug was mixed with 3% w/v PLGA 50:50 in dichloromethane (DCM, Millipore Sigma) and sonicated at 20 watts for 2 min to form an oil phase. This mixed solution was then added drop-by-drop to 15 mL of 5% w/v polyvinyl alcohol (PVA, Millipore Sigma) followed by emulsification for 10 min at 30 watts. To remove the solvent, the suspension was then stirred overnight, ultra-centrifuged, and lyophilized to collect the drug-loaded NPs.

The size, size distribution, and surface charges of newly prepared NPs were characterized using a ZetaPALS zeta potential analyzer (Brookhaven Instruments Inc.). Drug loading efficiency for these NPs was determined as described before^23^. Briefly, NPs were first dissolved in DCM to precipitate the polymer followed by addition of 100% ethanol. The mixture was then centrifuged to separate the polymer (in DCM, lower phase) and drug (in ethanol, upper phase). The amount of drug loaded into the NPs was measured by its absorbance at 380 nm using a UV-Vis spectrophotometer (Infinite M200 plate reader, Tecan, San Jose, CA) and determined by the following formula: The drug release kinetics of the NPs was measured as described before^23^. Briefly, PLGA-Comp34 NPs were resuspended in PBS (pH 7.4) at a 1 mg/ml concentration and added to a dialysis bag (100 kDa molecular weight cutoff, Spectrum Laboratories, Rancho Dominguez, CA) for up to 21 days at 37 °C. At each time point, 1 mL of the dialysates were collected and replenished with fresh PBS. The amount of drug released was determined by its absorbance at 380 nm using a UV-Vis spectrophotometer from a predetermined standard curve of comp34.

### Biotin pull-down assay and mass spectrum analysis

MDA-MB-231 cells were harvested and lysed in RIPA buffer supplemented with protease and phosphatase inhibitors cocktail (Prod#: 1862209; Prod#:1862495, Thermo Scientific). After centrifugation at 13,000 × *g* for 20 min, the supernatant was collected and equally divided into three parts, one of them was used as input; two of them were incubated with 100 µM of biotin or Comp34-biotin, respectively, and 100 µl of Streptavidin MagBeads (Cat#: L00424, GeneScript) in each tube overnight at 4 °C, according to the manufacturer’s instructions. After incubation, the beads were washed three times with lysis buffer, and the bead-bound proteins separated by SDS-PAGE, and visualized by silver staining for mass spectrometry (Cat#: 24600, ThermoFisher Scientific) according to the manufacturer’s instructions. The indicated band in the gel was excised, followed by in-gel digestion and analysis by mass spectrometry.

### Xenograft model of human breast cancer

For TNBC xenograft tumor models, MDA-MB-231 (5 × 10^6^) cancer cells were injected subcutaneously in the flank of female athymic nude mice (nu/nu, 4 weeks old) (The Jackson Laboratory, Bar Harbor, ME). Two weeks after the injection of cancer cells, xenograft tumors were found in the injection site in most mice. Tumors will be allowed to grow to an average of 100 mm^3^ prior to intraperitoneal injection of drugs. Animals were treated with vehicle (*n* = 10), rapamycin (5 mg/kg, i.p., *n* = 12), or Comp34 (5 mg/kg, i.p., *n* = 12). To improve aqueous solubility and bioavailability of Comp34, we developed poly(lactic-co-glycolic acid) nanoparticles (PLGA-NPs) of Comp34. The drug-loaded PLGA-NP size was 187 ± 61 nm with zeta potential of −18 mV. The drug loading efficiency was 62%, resulting in 85 µg of C23 in each mg of PLGA-NP. The in vitro time course of drug release kinetics was shown in Fig. S6D. The 100 μl Comp34 formulation or rapamycin was administered to mice once every two days intraperitoneally for 4 weeks. Tumor dimensions were measured twice a week, and tumor volumes of MDA-MB-231 xenografts were calculated by length (L), width (W), and height (H) using the formula (volume = π/6 × L × W × H). Mice health conditions were checked every day, and body weight was checked every 3 days. Mice were immediately euthanized if their tumor volume exceeded 2000 mm^3^ or the weight loss exceeded 20% of the initial body weight. At the end of the experiment, the animals were sacrificed and the tumors harvested. All animal studies were performed in accordance with the guidelines approved by the Institutional Animal Care and Use Committee of Charles Drew University of Medicine and Science.

### Statistical analysis

Statistical analysis in the present study was performed using GraphPad Prism version 7.00 software (GraphPad Software Inc). Data were presented as mean ± SD from at least three experiments and used two-tailed Student’s *t*-tests to detect differences. Correlation analysis were performed by Pearson’s correlation and spearman’s rank correlation. The effects of treatments on tumor size were evaluated using 2-way Analysis of Variance (ANOVA) and Fisher’s post-hoc test. A *P* value <0.05 was considered statistically significant.

## Results

### Comp34 preferentially targets TNBC and cancer stem cells and inhibits AKT1/mTOR expression

In spite of the exciting findings of curcumin against various cancer cells, the clinical application of curcumin in cancer treatment is limited because of its unacceptable therapeutic effects in vivo. Here, we designed and synthesized a series of analogs^24^ (Fig. S1A) and evaluated their antibreast cancer activity. Cell viability screening of 33 analogs shows that Comp34, Comp45, Comp39, Comp44, and Comp46 inhibit MDA-MB-231 cell growth (Fig. 1a). Among them, Comp34 (Fig. 1b) exhibits the lowest IC_50_ (63 nM). Compared to ER(+) BC cell lines MCF-7, T47D, and ZR-75-1, Comp34 shows more potent cytotoxicity against TNBC cells (Fig. 1c). Intriguingly, at these concentrations, Comp34 does not affect the viability of MCF-10A/MCF-12A cells. Thus, we focus on studying the anti-TNBC effect and the related molecular mechanism of Comp34 in the following work.

Next, to explore the molecular mechanisms of Comp34’s anticancer action, we employed a human phospho-antibody array to study a subset of phosphorylation events after Comp34 stimulation. Among 34 phospho-antibodies on the array, Comp34 treatment led to the decreased level of 5 phosphorylated proteins (p70 T389, TOR, STAT3, HSP60, AKT) and increased level of five phosphorylated proteins (STAT5a/b, FAK, c-Jun, HSP27, STAT6) compared with nonstimulated samples (Fig. 1d, e). Among them, the phospho-level of p70 T389 and TOR, which are involved in mTOR pathway, gained the greatest change, implying that they may be related to the anticancer action of Comp34. To substantiate this notion, we went on to validate these phosphorylation events by using Western analysis. Comp34-induced phosphorylation changes such as AKT1 and mTOR downstream targets (p70 S6 kinase, rpS6 and 4EBP1) confirmed the phospho-antibody array data (Fig. 1f). We also confirmed the main results by using MDA-MB-436 cells (Fig. S1C). Unexpectedly, AKT1 and mTOR expression at protein and mRNA levels are dramatically suppressed by Comp34 in a dose-dependent manner (Fig. 1f, g). This is different from the conventional AKT or mTOR inhibitors such as rapamycin, which only inhibit kinase activity but not protein expression. The AKT/mTOR signaling pathway plays a key role in cancer stem cells (CSCs) maintenance and viability in BC^25–28^. Remarkably, Comp34 shows profound effects on CSCs subpopulation in a dose-dependent manner (Fig. 1h, i). To investigate whether the observed reduction is specific, MDA-MB-231 cells were cultured with 100 nM Comp34 for 24 h followed by incubation in the media without Comp34. CD44^+^CD24^low+^ population cells reappear after withdrawal of this treatment, suggesting that Comp34 specifically affects CSCs phenotype (Fig. S1B).

### miR-99 family regulates AKT1 and mTOR in breast cancer cells

**Fig. 2:**
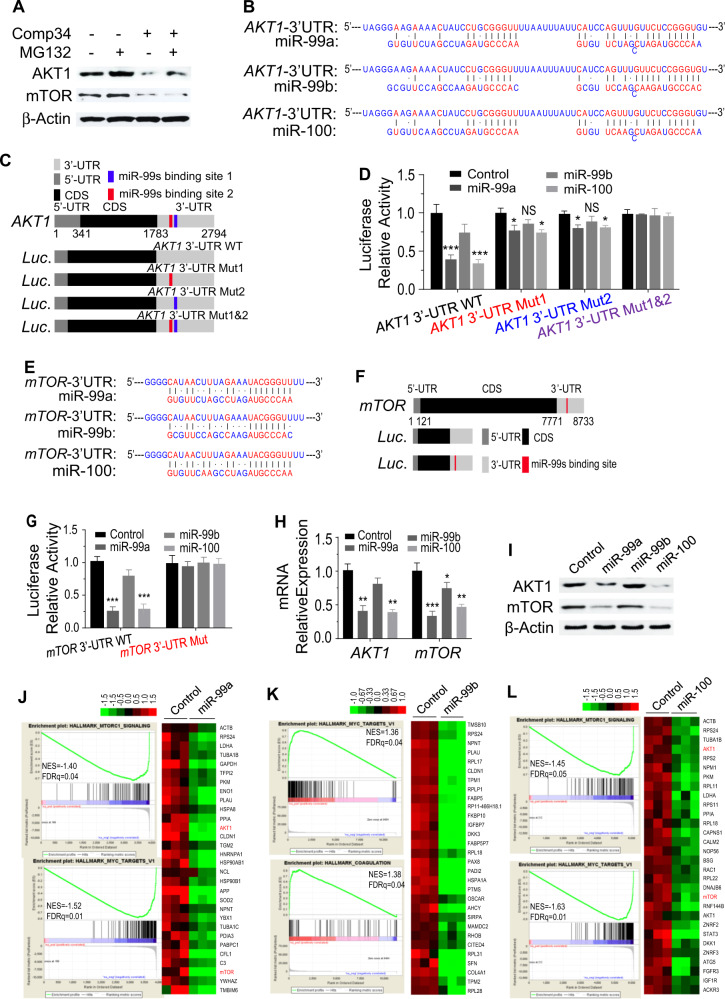
miR-99s transcriptionally regulates AKT1 and mTOR expression. WB analysis of the expression of AKT1 and mTOR in the MDA-MB-231 cells treated with Comp34 (100 nM), MG132 (1 mM), or their combination for 24 h. **b** Bioinformatics prediction of miR-99s binding site1 and site2 at two distinct positions in human *AKT1* 3′-UTRs by TargetScan. **c** A schematic representation of luciferase reporter of WT or consecutive mutation of *AKT1* constructs containing the 5’-UTR and CDS of luciferase, predicted targeting sequences and the corresponding mutants cloned into the 3’-UTR of luciferase. UTR Untranslated Region, CDS Coding Sequence. **d** miR-99s reduce the luciferase activity of wild-type (WT) instead of the mutated *AKT*1 3’-UTR. Data are presented as relative luciferase activity of firefly luciferase activity to *renilla* luciferase activity (*n* = 3). *P < 0.05 versus control; **P < 0.01 versus control. **e** Predicted miR-99s binding site in human *mTOR* 3’-UTR. **f** A schematic representation of luciferase reporter of WT or consecutive mutation of *mTOR* constructs. **g** miR-99s reduce the luciferase activity of WT instead of the mutated *mTOR* 3’-UTR. Data are presented as relative luciferase activity of firefly luciferase activity to *renilla* luciferase activity (*n* = 3). ***P < 0.001 versus control. **h**, **i** The effect of the miR-99s on the mRNA and protein expression of AKT1 and mTOR. MDA-MB-231 cells were transfected with miR-99s mimics or negative control. Real-time PCR and Western blot analysis were performed to measure the mRNA (**h**) and protein (**i**) levels of AKT1 and mTOR, respectively. *P < 0.05 versus control; **P < 0.01 versus control. Heatmaps representing the top 30 downregulated genes associated with overexpression of miR-99a (**j**), miR-99b (**k**), and miR-100 (**l**) in MDA-MB-231 cells.

We next asked whether the Comp34-induced decreases in protein levels was associated with proteasome pathway. Indeed, the treatment with the proteasome inhibitor MG132 does not change Comp34-induced protein decrease in MDA-MB-231 cells (Fig. 2a), suggesting that AKT1 and mTOR protein are not turned over through the ubiquitin-proteasome pathway. Because both the mRNA and protein level of AKT1 and mTOR were downregulated by Comp34, we reasoned that the regulation occurs at the transcription level. Bioinformatics analysis shows that the 3’-UTRs of *AKT1* and *mTOR* harbor multiple miR-99 family member binding sites (Fig. 2b, e). We speculated that the microRNAs regulation might involve this process. The 3’-UTR fragments of *AKT1* or *mTOR* was inserted downstream of a luciferase open reading frame (ORF) in a reporter gene plasmid to test whether they were directly repressed by the miR-99s (Fig. 2c, f). The 3’-UTRs of both *AKT1* and *mTOR* conferred repression of the heterologous luciferase ORF after transfection of miR-99a, miR-99b, or miR-100. Overexpression efficiency of miR-99a, miR-99b, and miR-100 are shown in Fig. S2A. The repression by the miR-99 family was abolished when the predicted target sites were mutated (Fig. 2d, g). Thus, the reduction of mRNA and protein expression of AKT1 and mTOR by the miR-99 family is either due to an indirect effect or due to a target site in the ORF. Then we tested whether the miR-99 family regulates the expression of the AKT1/mTOR. *AKT1* and *mTOR* mRNA levels were decreased upon transfection with miR-99a or miR-100 mimics instead of miR-99b (Fig. 2h). Reduction in the protein levels of AKT1 and mTOR were also observed in cells transfected with miR-99a or miR-100 mimics (Fig. 2i). The number of CD44^+^CD24^low+^ population cells is also decreased in MDA-MB-231 cells transfected with miR-99a or miR-100 mimics (Fig. S2B).

To understand the mechanisms whereby miR-99s regulate the pathways that control tumorigenesis of BC, a genome-wide unbiased approach to analyze gene expression by RNA sequencing was carried out in both miR-99s overexpression and control MDA-MB-231 cells. Bioinformatics analysis of the whole transcriptome revealed a profound impact on gene expression networks. Geneset enrichment analysis (GSEA) using the curated geneset compilation Hallmark (MSigDb H) of transcripts downregulated in the miR-99a group as compared with control group showed enrichment in gene sets corresponding to “mTORC1 signaling”, “EPITHELIAL_MESENCHYMAL_TRANSITION”, and “MYC targets_V1” are the top 3 enriched signatures (Figs. 2j, S2C). These results indicate that mTOR signaling genes, MYC target genes and EMT were repressed in MDA-MB-231 cells transfected with miR-99a. Further GSEA analysis using the curated geneset compilation Hallmark (MSigDb H) of transcripts downregulated in the miR-100 overexpression group demonstrated that “MYC targets_V1”, “mTORC1 signaling”, and “EPITHELIAL_MESENCHYMAL_TRANSITION” are also top three enriched signatures (Fig. S2E). The transcriptome profiling of miR-100 overexpression is highly similar to miR-99a in MDA-MB-231 cells (Figs. 2j, S2C). The heatmap shows the top 30 genes significantly downregulated by miR-99a or miR-100 overexpression (Fig. 2j, l). RNA sequencing data confirmed that *AKT1* and *mTOR* decrease in miR-99a or miR-100 transfected cells. However, miR-99b overexpression shows significantly distinct transcriptome profiling compared with miR-99a and miR-100. Most enriched gene signatures are “COAGULATION”, “MYC_TARGETS_V1”, and “EPITHELIAL_MESENCHYMAL_TRANSITION” with the curated geneset compilation Hallmark (MSigDb H) of upregulated transcripts. None of these gene sets are enriched in negative phenotype (Fig. S2D). Figure 2k shows top 30 significantly downregulated genes induced by overexpression of miR-99b. Together, AKT1 and mTOR are direct targets of the miR-99a and miR-100, but not miR-99b.

### Decreased miR-99s expression and elevated *AKT1* expression contribute to BC progression

**Fig. 3:**
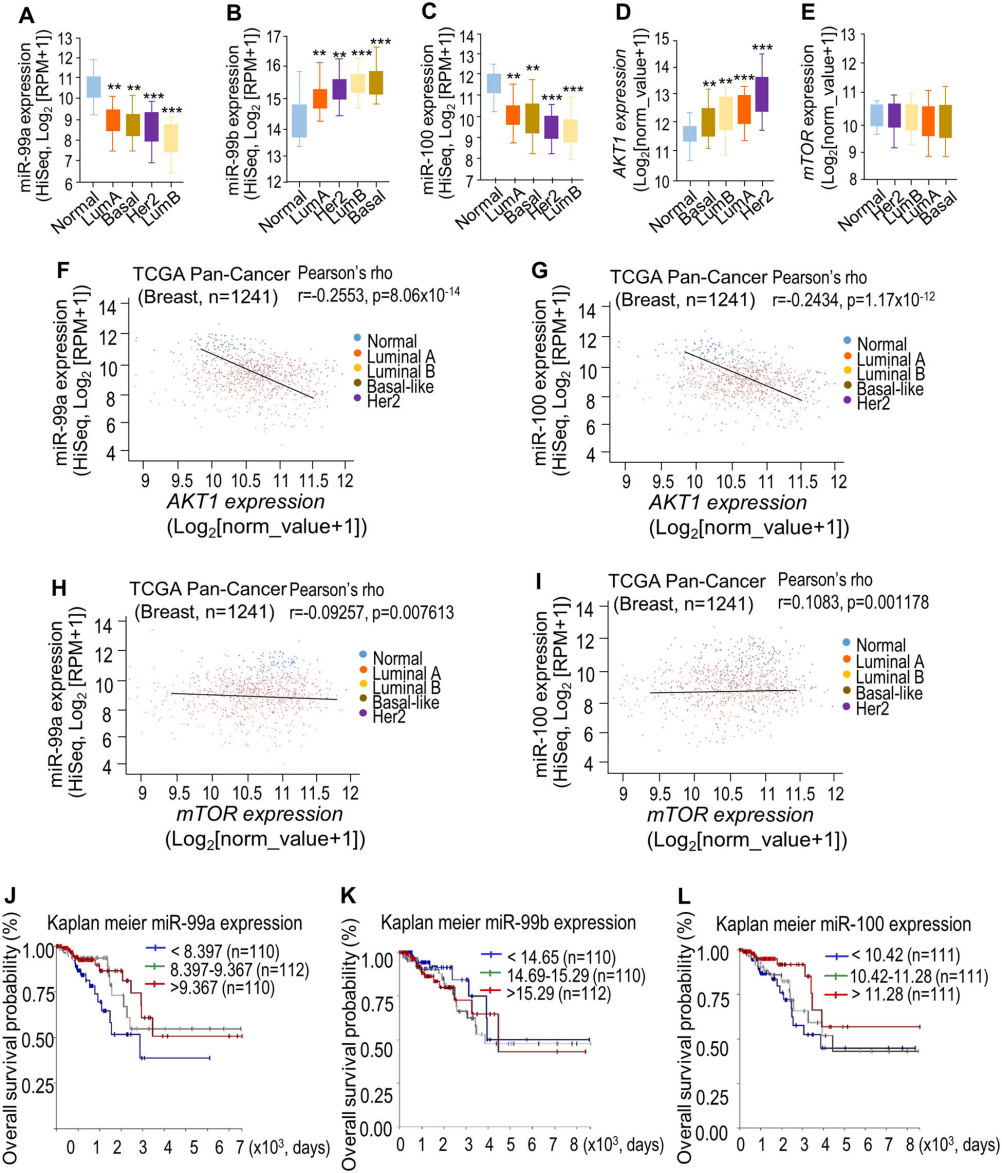
miR-99s and AKT1/mTOR expressions in breast cancer samples. **a–e** RNA-seq data for expression of miR-99s (**a**–**c**), *AKT1* (**d**), and *mTOR* (**e**) in the indicated subtype in The Cancer Genome Atlas (TCGA) database. Expression of AKT1 is negatively associated with the expression of miR-99a (**f**) and miR-100 (**g**) in breast cancer tissues in the TCGA PAN-CANCER breast database. The correlation between mTOR and miR-99a (**h**) or miR-100 (**i**). **j**–**l** Overall survival in patients with breast cancer stratified according to miR-99s expression status in their primary tumors. Survival time data are presented as means ± SEM. The log-rank test *P* value reflects the significance of the correlation between miR-99s positivity and longer survival outcome.

To better understand the clinical significance of our findings, we examined the signatures of miR-99s, *AKT1*, and *mTOR* in BC samples by using TCGA databases. RNA-seq data analysis by XENA revealed that miR-99a and miR-100 expression decreased by ~30–40% in all PAM50 tumor subtype compared with normal breast tissues (Fig. 3a, c). miR-99b is highly expressed in tumor samples (Fig. 3b). In contrast, *AKT1* expression in tumor samples is significantly higher than that in normal breast (Fig. 3d). However, *mTOR* expressions are slightly lower in tumor samples (Fig. 3e). Correlation analysis revealed that miR-99a and miR-100 are significantly negatively associated with *AKT1* expression (*p* = 8.06 × 10^−14^ and *p* = 1.17 × 10^−12^, respectively) (Fig. 3f, g) and that miR-99b is positively correlated with *AKT1* expression (*p* = 2.51 × 10^−11^) (Fig. S3A). These results confirm that miR-99a and miR-100, rather than miR-99b, can negatively regulate *AKT1* expression in cancer patients. There is no obvious correlation between the expression of miR-99s and *mTOR* (Figs. 3h, i, and S3B), which may be attributed to no significant change of *mTOR* expressions in BC samples. Furthermore, Kaplan–Meier survival analysis revealed that low expression of miR-99a/miR-100 and high expression of *AKT1* are associated with poorer survival rate of BC patients (Figs. 3j, l and S3C). The correlations between the expression of miR-99b, *mTOR*, and survival rate of BC patients are obscure (Figs. 3k and S3D). The trends of low survival rates related to low expression of miR-99a/b and high expression of *AKT1* demonstrate that miR-99a and miR-99b are anti-oncogenic and *AKT1* is a proto-oncogene.

### Screening of potential miR-99 family sponges

**Fig. 4:**
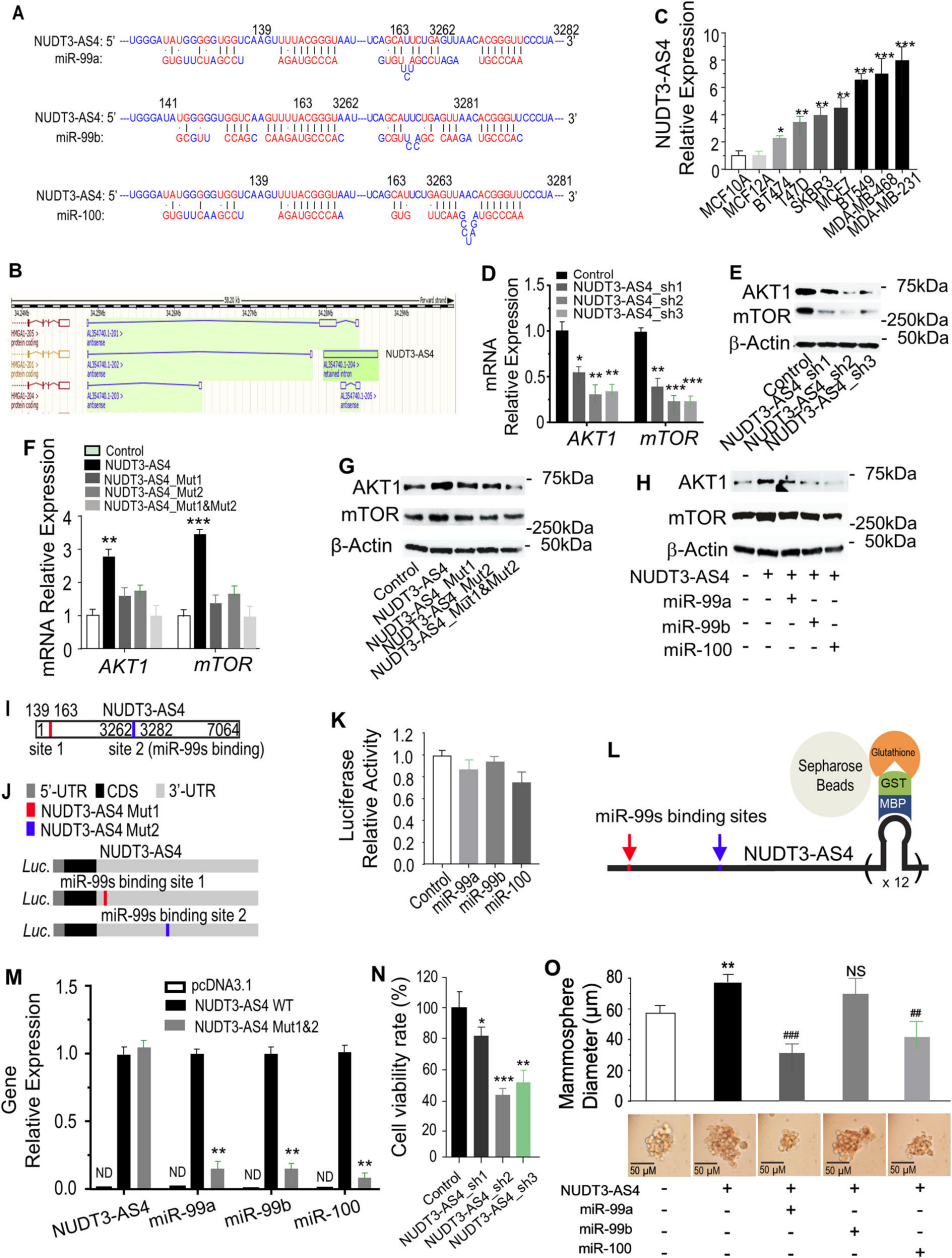
NUDT3-AS4 is physically associated with the miR-99s. **a** Bioinformatics prediction of miR-99s binding sites at two distinct positions in NUDT3-AS4. **b** Schematic diagram of NUDT3-AS4 isoforms from ENSEMBL annotation. **c** NUDT3-AS4 expression in breast cancer and nontumorigenic cell lines. **d** NUDT3-AS4 knockdown reduces mRNA (**d**) and protein (**e**) levels of AKT1 and mTOR. MDA-MB-231 cells were retrovirally transduced with NUDT3-AS4_sh or control. RT-PCR was performed to measure NUDT3-AS4, miR-99s, and the mRNA of *AKT1* and *mTOR* level, respectively. Immunoblots were performed to determine the protein levels of AKT1 and mTOR. **f**, **g** NUDT3-AS4 overexpression increases mRNA (**f**) and protein (**g**) levels of AKT1 and mTOR. MDA-MB-231 cells were transfected with NUDT3-AS4 WT or the respective mutants. RT-PCR and WB analysis were performed to measure the mRNA and protein level, respectively. **P < 0.01, ***P < 0.001 versus control. **h** miR-99s abolish NUDT3-AS4 caused protein levels increase of AKT1 and mTOR. **i** A schematic representation of NUDT3-AS4. The putative miR-99s binding sites in the NUDT3-AS4 are shown in red and blue boxes. **j** A schematic representation of luciferase reporter of WT or consecutive mutation of NUDT3-AS4 constructs. **k** miR-99s do not change the luciferase activity of NUDT3-AS4 WT. MDA-MB-231 were cotransfected with miR-99s and luciferase reporters containing NUDT3-AS4 WT or mutant transcript. Data are presented as relative luciferase activity of *Renilla* to Firefly luciferase luciferase activity (*n* = 3). **l** A schematic outline of the MS2-RIP strategy used to identify endogenous NUDT3-AS4 binding complex. **m** Endogenous association of microRNAs with NUDT3-AS4 after MS2-RIP. MDA-MB-231 cells were transiently transfected with GST-fused MS2 binding protein (GST-MS2BP) together with pTAG2B-MS2-12XH19 or pTAG2B-MS2-12X (empty vector; MS2). Total cell extracts were prepared 24 h after transfection and precipitated by glutathione-sepharose beads. RNA was purified and analyzed by RT-qPCR to detect transfected NUDT3-AS4 or endogenous miR-99s. **n** NUDT3-AS4 knockdown inhibits MDA-MB-231 cells proliferation. **o** Dissociation of mammospheres after transfection with NUDT3-AS4 and miR-99s mimics. The results represent the mean diameter of mammospheres ± SD, *n* = 6. *P < 0.05; **P < 0.01 versus NUDT3-AS4 negative; ^##^*P* < 0.01 versus NUDT3-AS4 positive; NS not significant.

In order to explore the molecular mechanism of lncRNA-miRNA-mRNA axis in cancer-related pathways, DIANA-LncBase v2 was used to predict the lncRNA-miRNA-mRNA associations^29^. DIANA-LncBase determined all the candidate lncRNAs that were involved in possible regulation of miR-99 family members at a score threshold of 0.7. With bioinformatics analysis, several lncRNAs were predicted to be able to bind all miR-99 family members concurrently. Four lncRNAs (NUDT3-AS4, XLOC_013308, RP11-81F13.2, PROX1-AS1) harbor one or two sequences that matches seed sequences in all three miR-99 family members; three lncRNAs (TTTY15, RP11-147L13.2, RP11-379L18.1) harbor a sequence that matches seed sequences in two miR-99 family members (Table S1). These findings suggest that there might be lncRNAs playing an important role in regulation of the miR-99 family. To validate the bioinformatics prediction, we analyzed the expression of 7 lncRNAs using quantitative real-time PCR and found that the expression of NUDT3-AS4 (Transcript ID: ENST00000429998.3) is the highest in MDA-MB-231 cells. There is no difference in the expression of all 7 lncRNAs in MCF-12A cells (Fig. S4A). NUDT3-AS4 expression were examined by qPCR. A gene-specific reverse transcript primer was used in the reverse transcript step to avoid amplify the NUDT3. NUDT3-AS4 was highly expressed in all BC cell lines, especially in TNBC cells (Fig. 4c). Therefore, we focused on NUDT3-AS4 and further studied its role in the antitumor effect of Comp34. The NUDT3-AS4 harbors multiple miR-99 family members’ binding sites (Fig. 4a). The AL354740.1 gene has five transcripts (Fig. 4b). NUDT3-AS4 is a novel transcript with a biotype of retained intron and a length of 7065 bps (Fig. 4i). To identify the role of NUDT3-AS4 in BC cell progression, we depleted NUDT3-AS4 gene expression by transducing NUDT3-AS4 shRNA lentiviral transduction particles into MDA-MB-231 cells. The knockdown efficiency was confirmed by qPCR (Fig. S5A). The expression of AKT1 and mTOR is decreased at both mRNA and protein levels after the transduction (Fig. 4d, e). Furthermore, NUDT3-AS4 WT or the respective mutant plasmids were transfected into MDA-MB-231 cells. NUDT3-AS4 WT overexpression increases the mRNA levels of *AKT1* and *mTOR* by 2.7- and 3.4-fold, respectively. However, NUDT3-AS4 mutations (Mut1/Mut2) at the miR-99s binding sites hardly changes AKT1/mTOR mRNA and protein expression (Fig. 4f, h), which proves NUDT3-AS4 regulates the AKT1 and mTOR expression by miR-99s binding. Consistently, the increased protein expression of AKT1 and mTOR upon NUDT3-AS4 overexpression are attenuated by miR-99s transfection (Fig. 4h). However, we did not find any difference in NUDT3-AS4 expression after overexpression of miR-99s (Fig. S4C, D). Ectopically expressed NUDT3-AS4 WT or the mutant also does not change the levels of miR-99s (Fig. S4G). For further confirmation, we constructed luciferase reporters containing the full length of NUDT3-AS4, which contains wild-type (WT) or mutated miR-99s binding sites (Fig. 4j). Overexpression of miR-99s does not change the luciferase activities of both the WT (Fig. 4k) and mutant reporter vector (Fig. S4E, F). LncRNAs have diverse subcellular localization patterns. Here, we used single molecule, single-cell RNA fluorescence in situ hybridization (RNA FISH) to survey abundance and cellular localization patterns of NUDT3-AS4. We observed that the NUDT3-AS4 is almost exclusively localized in the cytoplasm. Simultaneous abundance analysis of NUDT3-AS4 shows no significant abundance change of NUDT3-AS4 upon transfection with miR-99s mimics (Fig. S4D). These data demonstrate that miR-99s bind to NUDT3-AS4 but do not induce its degradation. To validate the direct binding between miR-99s and NUDT3-AS4 at endogenous levels, MS2-RNA immunoprecipitation (RIP) to pull down endogenous microRNAs associated with NUDT3-AS4 (Fig. 4l) were performed, and demonstrated via qPCR analysis that miR-99s are enriched in the NUDT3-AS4 WT MS2-RIP compared to MS2-RIP of NUDT3-AS4 with mutated miR-99s targeting sites. The miR-99s cannot be detected in the empty vector group (pcDNA3.1) (Fig. 4m). Importantly, cell viability assay shows knockdown of NUDT3-AS4 inhibits MDA-MB-231 cells growth (Fig. 4n), and tumorsphere formation assay shows clear diminution of tumorsphere by miR-99s overexpression (Fig. 4o).

### Comp34 regulated AKT1 and mTOR expression through NUDT3-AS4

**Fig. 5:**
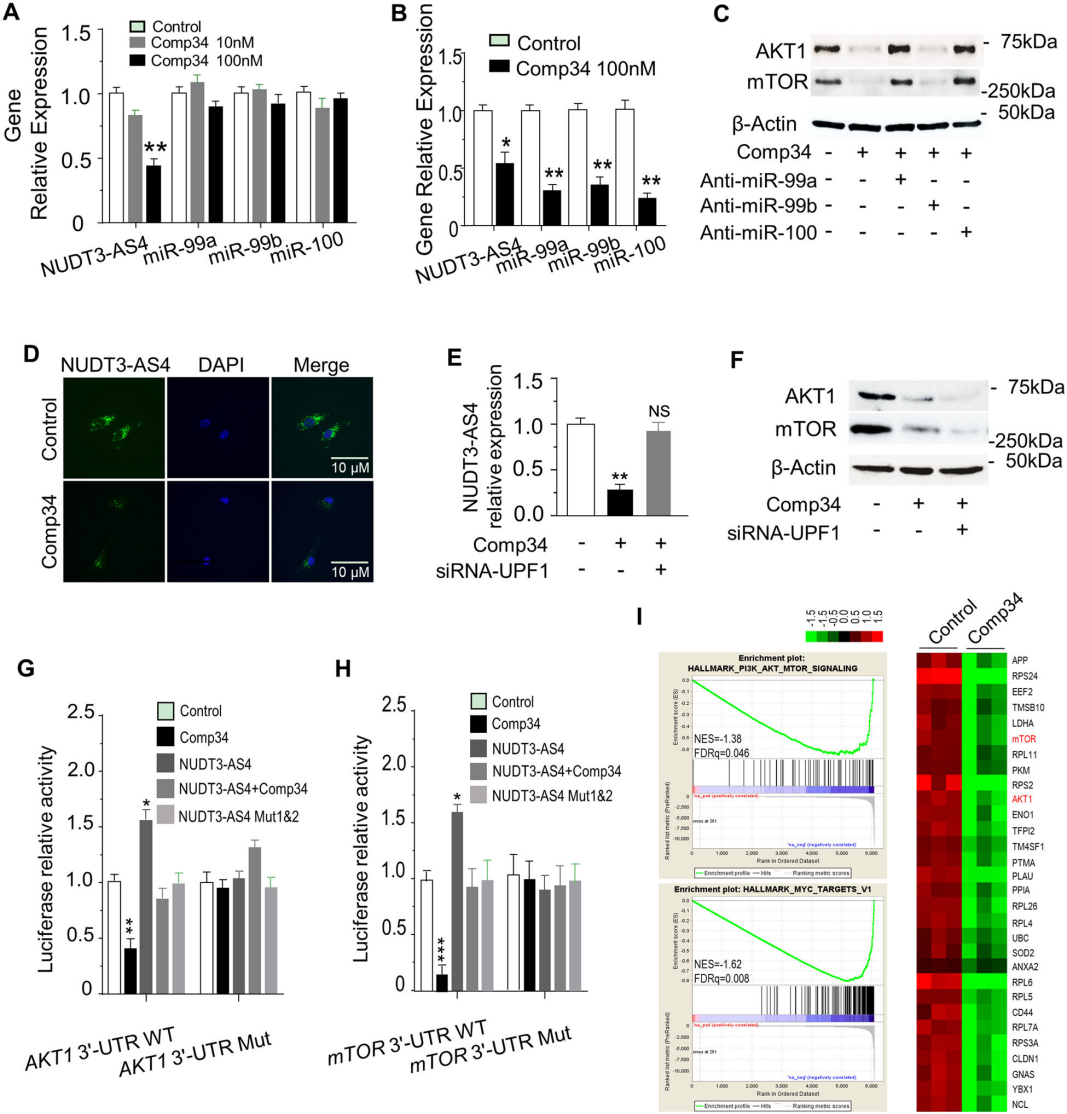
Comp34 regulates NUDT3-AS4/miR-99s/AKT/mTOR axis. **a** Comp34 decreases NUDT3-AS4, but not miR-99s expression. Quantitative RT-PCR and RNA fish (**d**) assays were performed to measure NUDT3-AS4 and miR-99s level. **P < 0.01 versus control. **b** Comp34 dissociates miR-99s from NUDT3-AS4 MS2-RIP complex. MDA-MB-231 cells were transiently transfected with GST-fused MS2 binding protein (GST-MS2BP) together with pTAG2B-MS2-12XH19 (in which NUDT3-AS4 was tagged with MS2-RNA hairpins). Endogenous miR-99s associated with NUDT3-AS4 was detected by MS2-RIP and qRT-PCR. *P < 0.05, **P < 0.01 versus control. **c** Anti-miR-99s rescue Comp34-induced decrease in AKT1 and mTOR protein levels. **d** Localization and abundance analysis of NUDT3-AS4 upon Comp34 treatment at single cell and single molecule resolution. **e** MDA-MB-231 cells were treated with a control siRNA or with a siRNA targeting UPF1. The relative expression levels of NUDT3-AS4 were determined by RT-qPCR. **P < 0.01 versus control; NS not significant. **f** AKT1 and mTOR protein expression levels were analyzed by immunoblot analysis. **g** Comp34 reduces, and NUDT3-AS4 increases the luciferase activity of AKT1 3’-UTR WT and mTOR 3’-UTR WT (**h**). Dual-luciferase reporter assays were performed to test the effect of Comp34 and NUDT3-AS4 on the AKT1 3’-UTR or mTOR 3’-UTR using the reporter gene constructs. Data are presented as relative luciferase activity of Renilla activity to Firefly luciferase luciferase activity (*n* = 3). **P < 0.01, ***P < 0.001 versus control. **i** GSEA plot of enrichment in “mTORC1_SIGNALING”, “MYC_TARGETS_V1” signatures in Comp34 treatment group versus control group (*n* = 3).

Comp34 decreases the expression of NUDT3-AS4, but does not change the expression level of miR-99s (Fig. 5a). We reasoned that Comp34 leads to miR-99s disassociation from NUDT3-AS4. To validate this notion, we performed an MS2-RNA immunoprecipitation (RIP) to pull down the exogenous NUDT3-AS4 that associates with the endogenous microRNAs, and demonstrated that miR-99s levels are significantly decreased in response to Comp34 treatment (Fig. 5b). Consistently, the decrease of AKT1 and mTOR protein expression upon Comp34 stimulation can be rescued by anti-miR-99s treatment (Fig. 5c).

Upon Comp34 stimulation, low abundance of NUDT3-AS4 is observed in bulk cell populations (Fig. 5d). To examine whether NUDT3-AS4 transcript is degraded by the nonsense-mediated RNA decay (NMD) pathway, we determined the abundance of NUDT3-AS4 upon Comp34 stimulation under treatment with NMD pathway blockade. Depletion of UPF1 attenuated Comp34-caused NUDT3-AS4 degradation (Fig. 5e), indicating that the NMD pathway is indispensable to NUDT3-AS4 degradation. Next, we raised the question of whether the dissociation of miR-99s from NUDT3-AS4 leads to the instability and degradation of NUDT3-AS4, or the degradation of NUDT3-AS4 occurs before miR-99s dissociation from NUDT3-AS4. As shown in Fig. 5f, the decrease in AKT1/mTOR protein levels caused by Comp34 was not inhibited by depletion of UPF1, implying that the answer is the former. Knockdown of UPF1 expression after siRNA transfection in MDA-MB-231 cells was confirmed by RT-PCR and Western analysis (Fig. S5A, B). Together, the mechanisms underlying Comp34 inhibition of lncRNA-NUDT3-AS4 involve Comp34-induced miR-99s dissociation from NUDT3-AS4 resulting in the instability and degradation of NUDT3-AS4 through the NMD pathway.

To substantiate the action mechanism of Comp34, we demonstrated that Comp34 reduces and NUDT3-AS4 increases the luciferase activity in WT *AKT1* and *mTOR* 3’-UTR constructs. Repression of luciferase activity by Comp34 is rescued by NUDT3-AS4 transfection, and the mutations in the putative corresponding miR-99s binding sites of NUDT3-AS4 do not affect the luciferase activity of *AKT1* and *mTOR* 3’-UTR (Fig. 5g, h). NUDT3-AS4-induced increase in luciferase activity is abolished by miR-99s (Fig. S5C, D). To further verify the downstream pathways regulated by Comp34, gene expression by RNA sequencing in both Comp34-treated and control cells was analyzed. GSEA analysis revealed that enrichment in gene sets corresponding to “mTORC1 signaling”, “MYC targets_V1”, and “EPITHELIAL_MESENCHYMAL_TRANSITION” are the top 3 enriched signatures (Fig. S5E). The transcriptome profiling of Comp34 treatment is highly consistent with miR-99a or miR-100 overexpression in MDA-MB-231 cells (Fig. S2C, E), further proving the Comp34’s role in inhibiting BC cell growth through miR-99s. The top 30 significantly downregulated genes upon Comp34 treatment are shown in a heatmap (Fig. 5i). RNA sequencing data confirmed the decrease of *AKT1* and *mTOR* in MDA-MB-231 cells treated by Comp34. (Fig. 5i). These results confirm that Comp34 inhibits the growth of BC cells through NUDT3-AS4 and miR-99s.

### The mechanism underlying Comp34 inhibition of mTOR is distinct from rapamycin

**Fig. 6:**
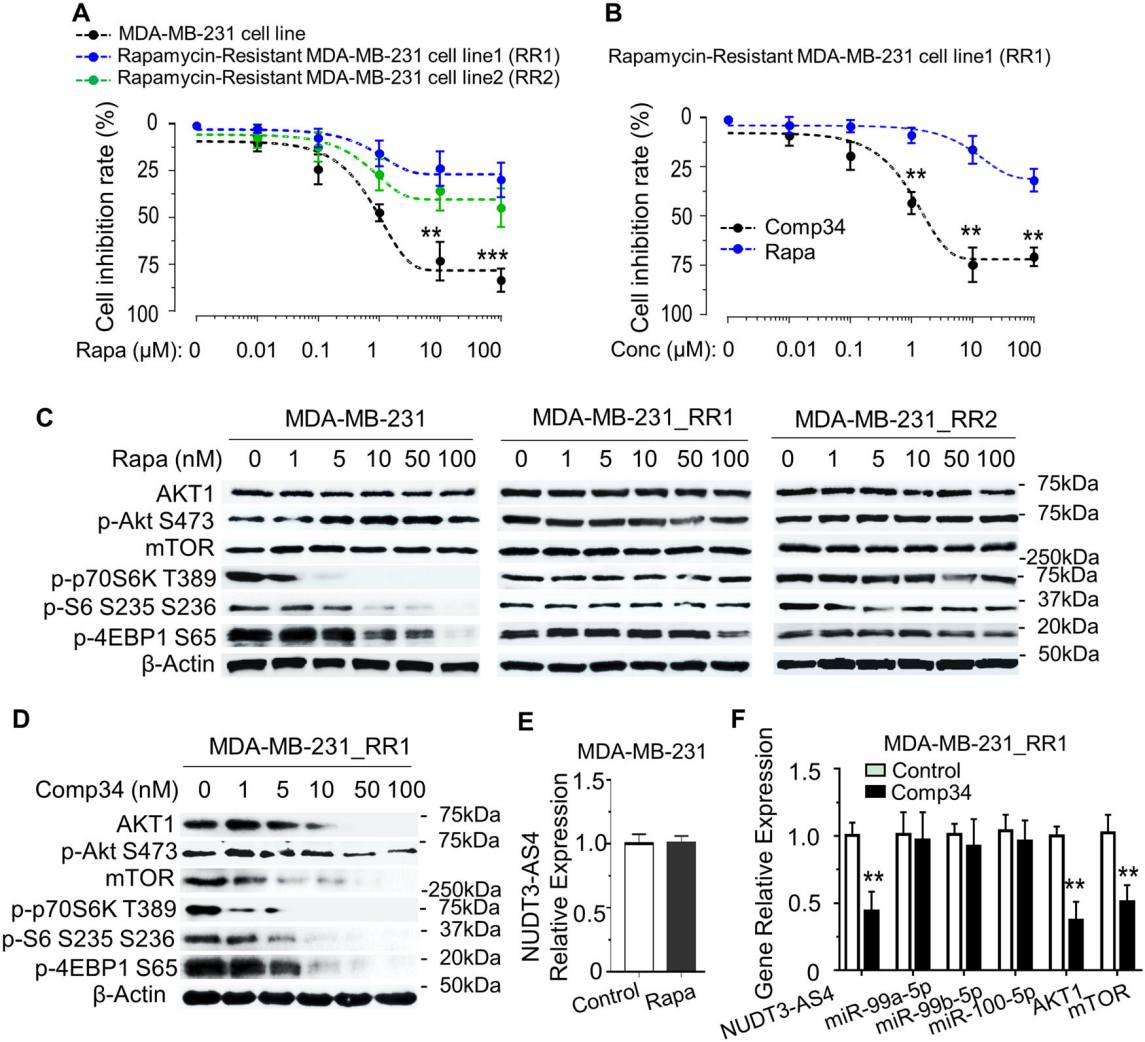
Comp34 targets and inhibits mTOR through cellular mechanisms different from rapamycin. **a** MDA-MB-231 and rapamycin-resistant MDA-MB-231 cell lines (RR1, RR2) were treated with the indicated concentrations of rapamycin for 48 h. MTS cell proliferation assay was performed to determine the cell growth inhibition. Each dot and error bar on the curves represents mean ± SD (*n* = 6). **b** RR1 cells were treated with Comp34 or rapamycin for 48 h. The cell growth inhibition was determined. Each dot and error bar on the curves represents mean ± SD (*n* = 6). **c** AKT/mTOR signaling was assessed by WB using the indicated protein-specific antibodies. **d** RR1 cells were treated with the indicated concentrations of Comp34 for 24 h. AKT/mTOR signaling was evaluated by WB. **e** MDA-MB-231 cells were treated with 100 nM rapamycin for 24 h. RT-PCR was performed to measure NUDT3-AS4 level. **f** RR1 cells were treated with or without 100 nM Comp34 for 24 h. RT-PCR was performed to measure NUDT3-AS4, miR-99s levels, and *AKT1/mTOR* mRNA levels. **P < 0.01 versus control.

To explore the mechanisms of mTOR inhibition by Comp34, we developed two rapamycin-resistant MDA-MB-231 cell lines (RR1 and RR2) and treated them with increasing concentrations of rapamycin or Comp34. Rapamycin inhibits cell growth of the parental cells, but not RR1/RR2 cells, in a dose-dependent manner (Fig. 6a). However, Comp34 has a significant inhibitory effect on RR1 compared to rapamycin (Fig. 6b), suggesting that they have different mechanisms of action. Rapamycin reduces phosphorylation of p70S6K (T389), S6 (S235/236) and 4EBP1 (S65) in MDA-MB-231 but not RR1 and RR2 cells, without affecting AKT1/mTOR protein expression (Fig. 6c). Intriguingly, Comp34 can not only inhibit the phosphorylation of effectors downstream of mTOR, but also significantly inhibit AKT1/mTOR protein expression in RR1 (Fig. 6d). Mechanically, Comp34 suppresses NUDT3-AS4 expression in MDA-MB-231 cells (Fig. 5a), but rapamycin has no such effects (Fig. 6e). Furthermore, in RR1 cells, Comp34 exhibits potent inhibitory effects on NUDT3-AS4 and AKT1/mTOR expression (Fig. 6f), which are fully mirrored by its impressive cytotoxicity in these cell lines. Consistent with the RR1 cell lines, similar results were observed by using another drug resistant cell line RR2 (Fig. S6). Together, Comp34 inhibition of mTOR is through a unique mechanism obviously different from rapamycin, namely suppression of lncRNA-NUDT3-AS4 expression, disassociation of miR-99s from NUDT3-AS4, and inhibition of AKT1/mTOR expression.

### Comp34 impairs the NUDT3-AS4-miR-99s complex

To identify the direct targets of Comp34, a pull-down assay was performed using Biotin-Comp34, followed by mass spectrometry analysis (Fig. S7B). Biotin was linked to Comp34 with a linker to generate biotin tagged Comp34 (Biotin-Comp34) (Fig. S7A), which retained its inhibitory effect on proliferation of MDA-MB-231 cells (Fig. S7G). Biotin-Comp34 or free biotin was incubated with cell lysate. Subsequently, the complexes were precipitated and purified using streptavidin-coated agarose beads followed by gel electrophoresis and silver staining. Multiple protein bands were precipitated by Biotin-Comp34 but not by free biotin (Fig. S7C). The resultant bands were excised and analyzed by mass spectrum (Fig. S7C). Immunoblotting data further confirm that SND1, HSPA8, CCT6A, PHB2, HSPB1 are binding proteins of Comp34 (Fig. S7D). Notably, these bands were competed away in the presence of free Comp34 at higher concentrations (Fig. S7D), indicating that Comp34 and Biotin-Comp34 bind to the same protein at the same site. To validate the endogenous binding RNA of Comp34, qPCR analysis of precipitated complex demonstrated that NUDT3-AS4 and miR-99s were significantly enriched in the Biotin-Comp34 binding complex, and that the affinity was competed away in the presence of free Comp34 at higher concentrations (Fig. S7E, F). NUDT3-AS4 and miR-99s could not be detected in the free biotin group. These results, in combination with the aforementioned data that Comp34 decreases the expression of NUDT3-AS4 (Fig. 5a, b) and significantly removes miR-99s away from NUDT3-AS4 (Fig. 5f), suggest that Comp34 directly targets NUDT3-AS4 and miR-99s, which elicits disassociation of the NUDT3-AS4/miR-99s complex, leading to subsequent instability and degradation of NUDT3-AS4 and release of miR-99s.

### Comp34 suppresses TNBCs in vivo

**Fig. 7:**
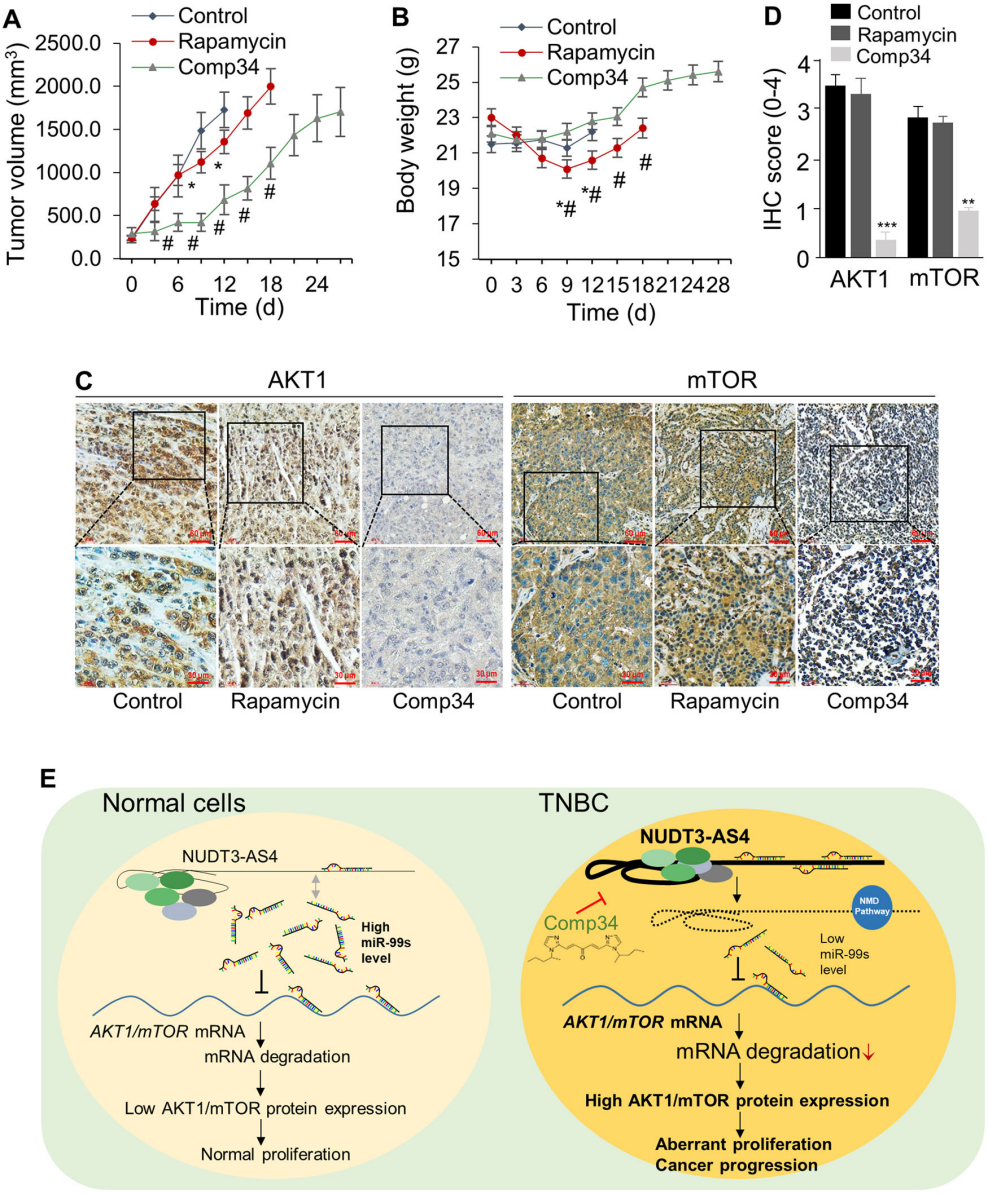
Effect of Comp34 on tumor growth in vivo. **a** Tumor volume of MDA-MB-231 cells transplanted into female athymic nude mice (nu/nu) and treated with vehicle (*n* = 10), rapamycin (5 mg/kg, i.p., *n* = 12), or Comp34 (5 mg/kg, i.p., *n* = 12) once every 2 days for 4 weeks. **b** body weight was checked every 3 days. *P < 0.05 versus control; ^#^*P* < 0.05 versus rapamycin. **c** Paraffin sections of xenograft tumor tissues were subjected to immunohistochemical (IHC) staining using the antibodies indicated. **d** Staining score of IHC analysis of AKT1 and mTOR. *p* value was calculated by one-way ANOVA. ***p < 0.001 vs control. **e** The identified signaling pathway in this study indicated that Comp34 represses TNBC growth via inhibition of AKT1/mTOR mRNA and protein expression. Long noncoding RNA (lncRNA) -NUDT3-AS4 acts as a microRNA sponge to compete with *AKT1/mTOR* mRNAs for binding to miR-99s, leading to decrease in degradation of *AKT1/mTOR* mRNAs and subsequent increase in AKT1/mTOR protein expression, which promotes TNBC growth and progression. Comp34-induced miR-99s dissociation from NUDT3-AS4 and resultant degradation of NUDT3-AS4 through the NMD pathway lead to increase in miR-99s-dependent degradation of *AKT1/mTOR* mRNA in TNBCs, subsequently inhibiting AKT1/mTOR protein expression and TNBC development.

Next, to determine Comp34 and rapamycin’s effects on tumor growth, MDA-MB-231 cells were subcutaneously injected into mammary fat pads of female athymic nude mice. Injections of DMSO, Comp34 or rapamycin (5 mg/kg, once every two days) were intraperitoneally given to the animals for 4 weeks (Fig. S7H). A statistically significant lower mean tumor volume was observed on as early as day 6 after injection in the mice administered with Comp34 than in the control mice (Fig. 7a), with no body weight loss observed (Fig. 7b). These results suggest that Comp34 displays a greater antitumor activity and lower toxicity than rapamycin in vivo. Its inhibitory effects on target proteins were substantiated by IHC analyses in xenograft tumors (Fig. 7c, d). This demonstrates that TNBC xenograft model is sensitive to the growth-inhibitory effect of Comp34.

## Discussion

lncRNAs have emerged as key players in different cellular processes and may exhibit tumor-suppressive and -promoting functions. They are expected to become novel biomarkers and therapeutic targets for cancer due to their genome-wide expression patterns in various tissues and their tissue-specific expression features. It is well known that cellular miRNA amount can be titrated for precise regulatory effects through artificial transcripts (miRNA sponges) containing tandem repeats of miRNA-binding sites^30^ and that lncRNAs may function as ceRNA^8,31,32^. Here, we demonstrate that lncRNA-NUDT3-AS4 acts as a microRNA sponge to compete miR-99s with *AKT1/mTOR* mRNAs, leading to decrease in degradation of *AKT1/mTOR* mRNAs and subsequent increase in AKT1/mTOR protein expression.

Our newly developed Comp34 affects transcripts and proteins of AKT1 and mTOR, and their downstream signaling cascades. We demonstrated that the 3’-UTRs of *AKT1* and *mTOR* harbor multiple miR-99 family member binding sites (Fig. 2b, e) and reasoned that the regulation occurs at the post-transcription level and miR-99s may mediate the Comp34-induced decrease in *AKT1/mTOR* mRNA. *AKT1*, and *mTOR* mRNA are direct targets of the miR-99a and miR-100 instead of miR-99b in TNBC. miR-99a and miR-100 have similar effect on the AKT1/mTOR expression and that overexpression of miR-99a and miR-100 has highly similar transcriptome profiling. These results can be elucidated by the highly similar nucleotides between miR-99a and miR-100. Compared with miR-99a and miR-100, miR-99b has four different nucleotides (Fig. S4H). This structure basis dictates the distinct biological function of the three miR-99 family members. Together, Comp34 induces decreases in the expression of NUDT3-AS4 and miR-99s disassociation from NUDT3-AS4, ultimately leading to decrease in *AKT1*/*mTOR* expression and inhibition of TNBC growth.

The mechanism underlying mTOR inhibition caused by Comp34 is different from the traditional kinase activity inhibitors such as rapamycin. Moreover, Comp34 displays a greater in vivo antitumor activity and lower toxicity than rapamycin. This may provide a new insight on the treatment of rapamycin nonsensitive BC patients or novel therapy targets on TNBC. The use of compounds inhibiting against AKT1/mTOR kinase protein has several advantages over kinase activity inhibition alone: (i) Targeting both AKT1 and mTOR protein expression has the theoretical advantage of hindering the feedback loop leading to mTORC2-induced AKT activation upon suppression of mTORC1^33,34^. (ii) Inhibition of kinase activity will always lead to feedback compensation increase in cellular protein expression of the kinases, eventually causing drug failure or drug resistance. (iii) Contrary to inhibition of the kinase activity, kinase protein inhibition is a strategy that may elicit a more thorough and lasting inactivation of downstream signals and avoid the “kinome rewiring” problem, whereby suppression of specific kinase activity results in compensatory feedback activation through alternative kinases^35,36^. In fact, the temporary elimination of kinase would prevent inactive kinases from continuing to act as scaffolding nodes, thereby restoring downstream carcinogenic signals^35,37–39^. Accordingly, we demonstrate that kinase protein inhibition leads to stronger suppression of cell proliferation and a more robust and persistent downstream signaling response. (iv) Small-molecule-mediated inhibition of the kinase protein itself rather than repression of the kinase domain would provide advantages e.g., decreased drug exposure time required to repress signaling and the capability to target kinase-independent functions. Together, the discovery of such a small-molecule compound capable of inhibiting AKT/mTOR pathway promotes the study of these key proteins in normal and carcinogenic signals, and also assists in developing more effective therapeutic drugs for AKT/mTOR-related diseases, particularly TNBC.

In conclusion, LncRNA-NUDT3-AS4 may play a proproliferative role in TNBC and be considered a relevant therapeutic target. Comp34 presents promising activity as a single agent to inhibit TNBC growth through inhibition of NUDT3-AS4, which functions as a sponge absorbing miR-99s to release the mRNA of *AKT1* and *mTOR* from miR-99s-dependent decay (Fig. 7e). Our findings provide convincing rationale for the further development of Comp34 as a TNBC-targeting agent.

## Supplementary information

Supplemental Figure 1

Supplemental Figure 2

Supplemental Figure 3

Supplemental Figure 4

Supplemental Figure 5

Supplemental Figure 6

Supplemental Figure 7

Supplemental tables

Supplementary Figure Legends
