# Supplementary figures and images for: Comp34 displays potent preclinical antitumor efficacy in triple-negative breast cancer via inhibition of NUDT3-AS4, a novel oncogenic long noncoding RNA
Source: Cell Death Dis. 2020 Dec 11;11(12):1052. doi: 10.1038/s41419-020-03235-w (PMC7733521; doi:10.1038/s41419-020-03235-w)

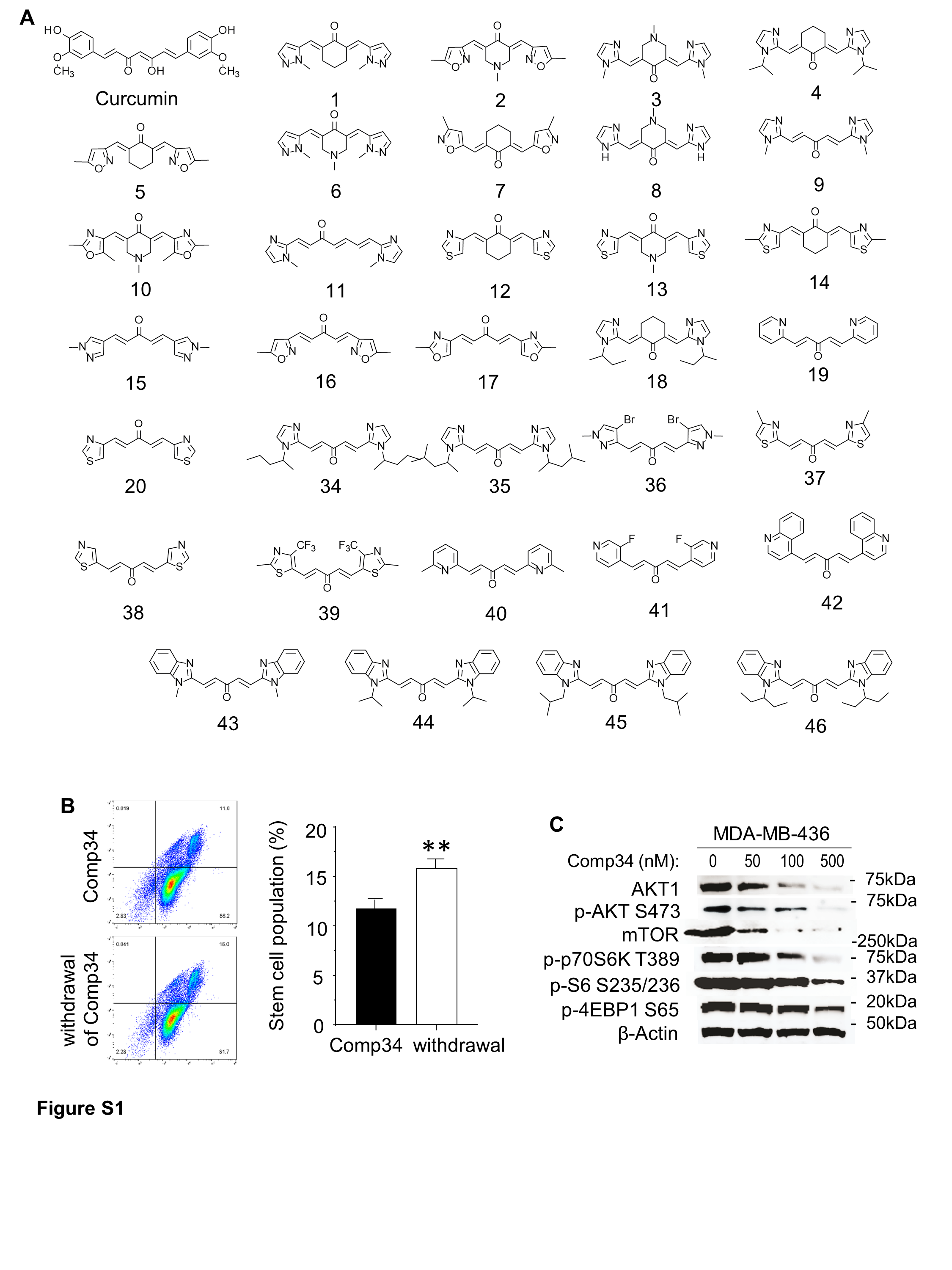

Supplement: Supplementary file 1 — Supplemental Figure 1 [file 41419_2020_3235_MOESM1_ESM.tif]

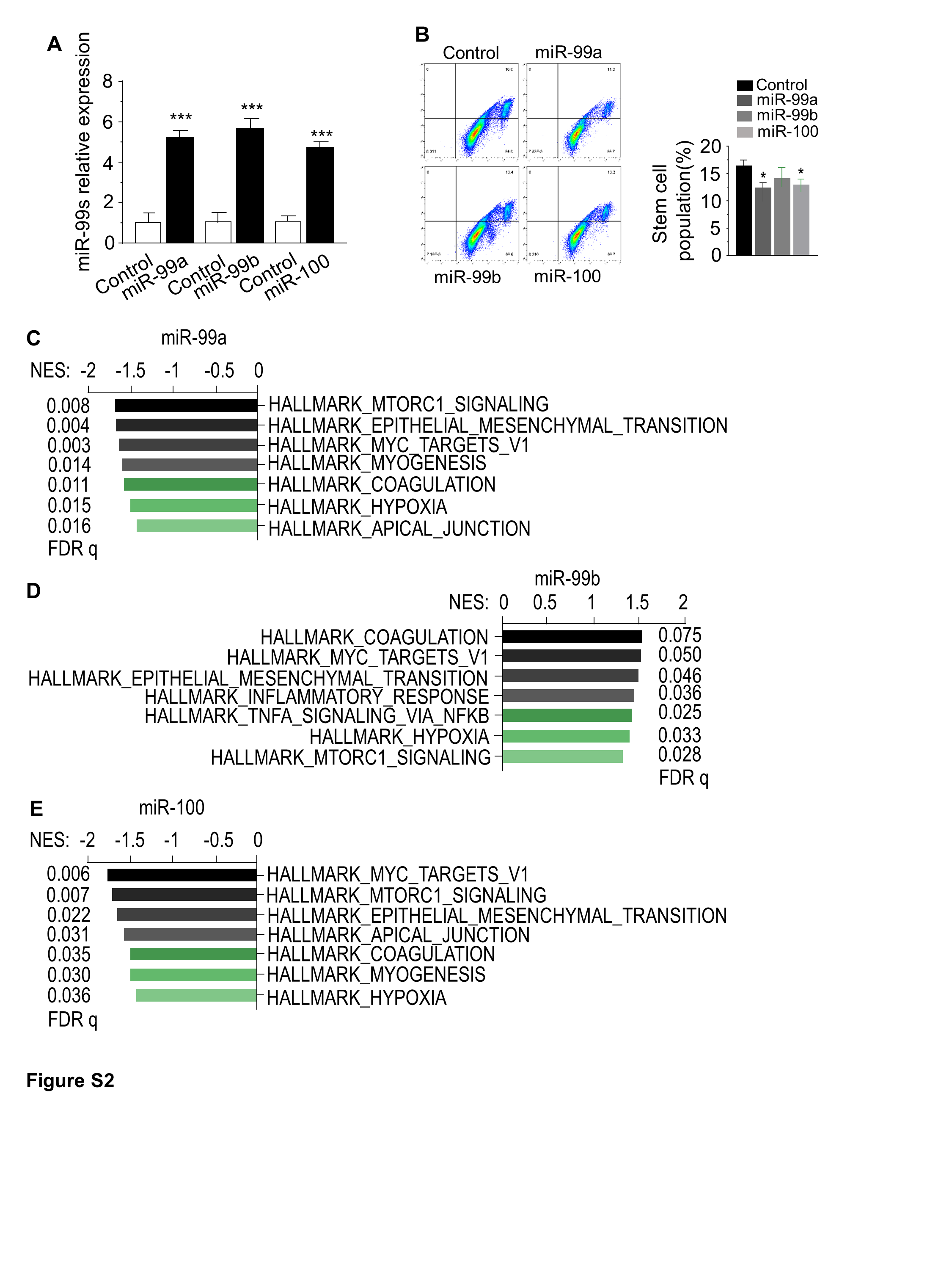

Supplement: Supplementary file 2 — Supplemental Figure 2 [file 41419_2020_3235_MOESM2_ESM.tif]

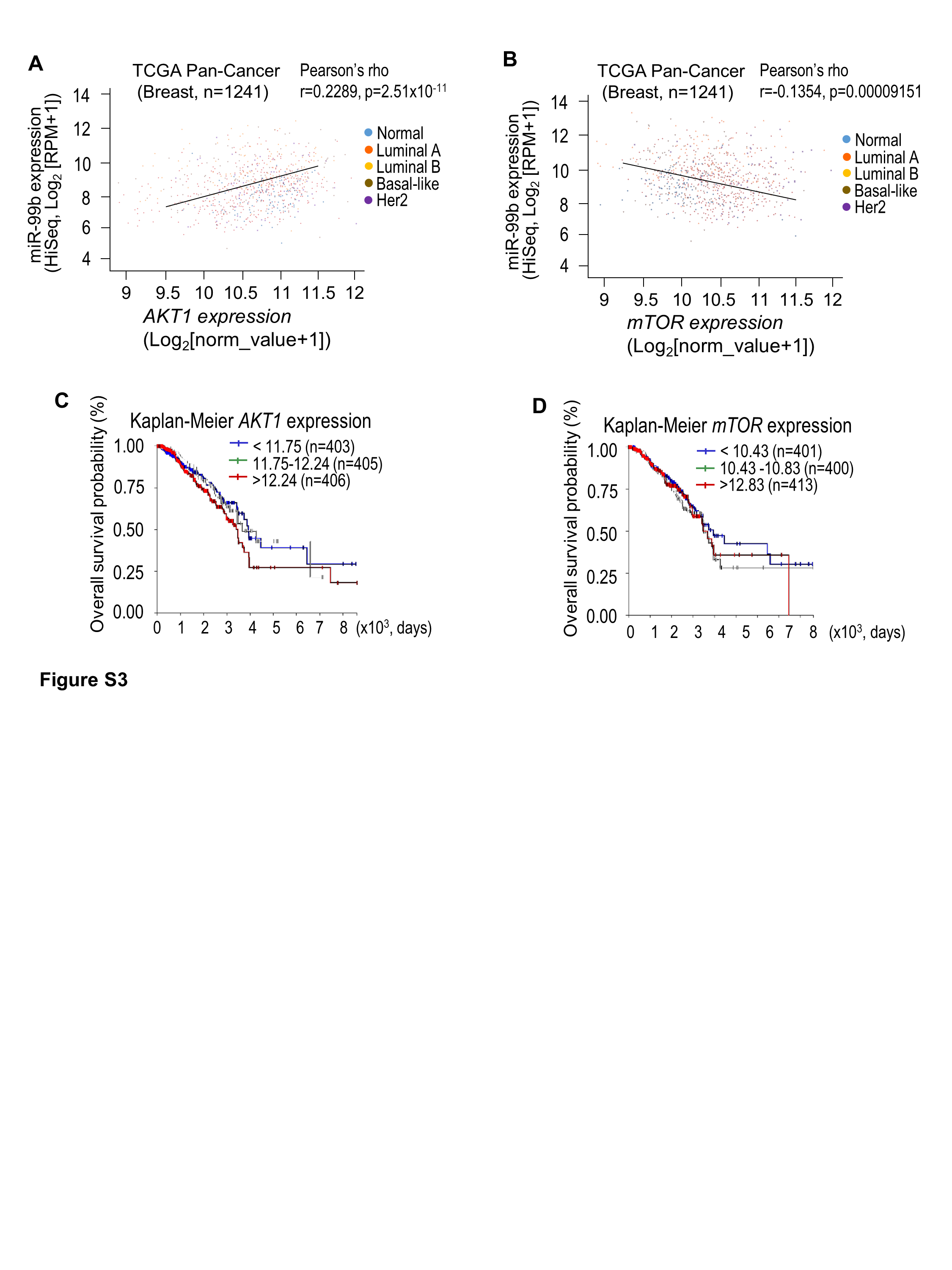

Supplement: Supplementary file 3 — Supplemental Figure 3 [file 41419_2020_3235_MOESM3_ESM.tif]

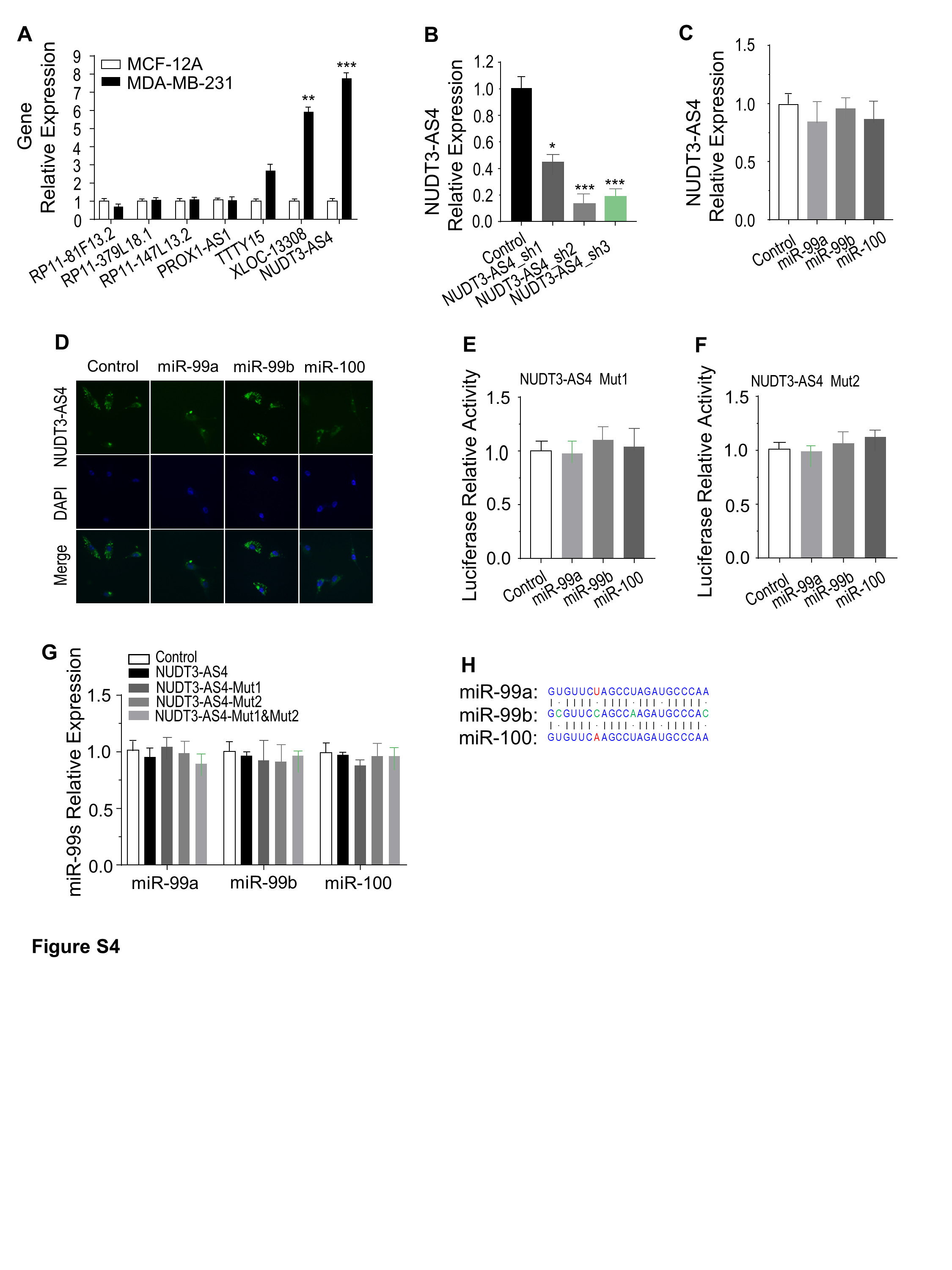

Supplement: Supplementary file 4 — Supplemental Figure 4 [file 41419_2020_3235_MOESM4_ESM.tif]

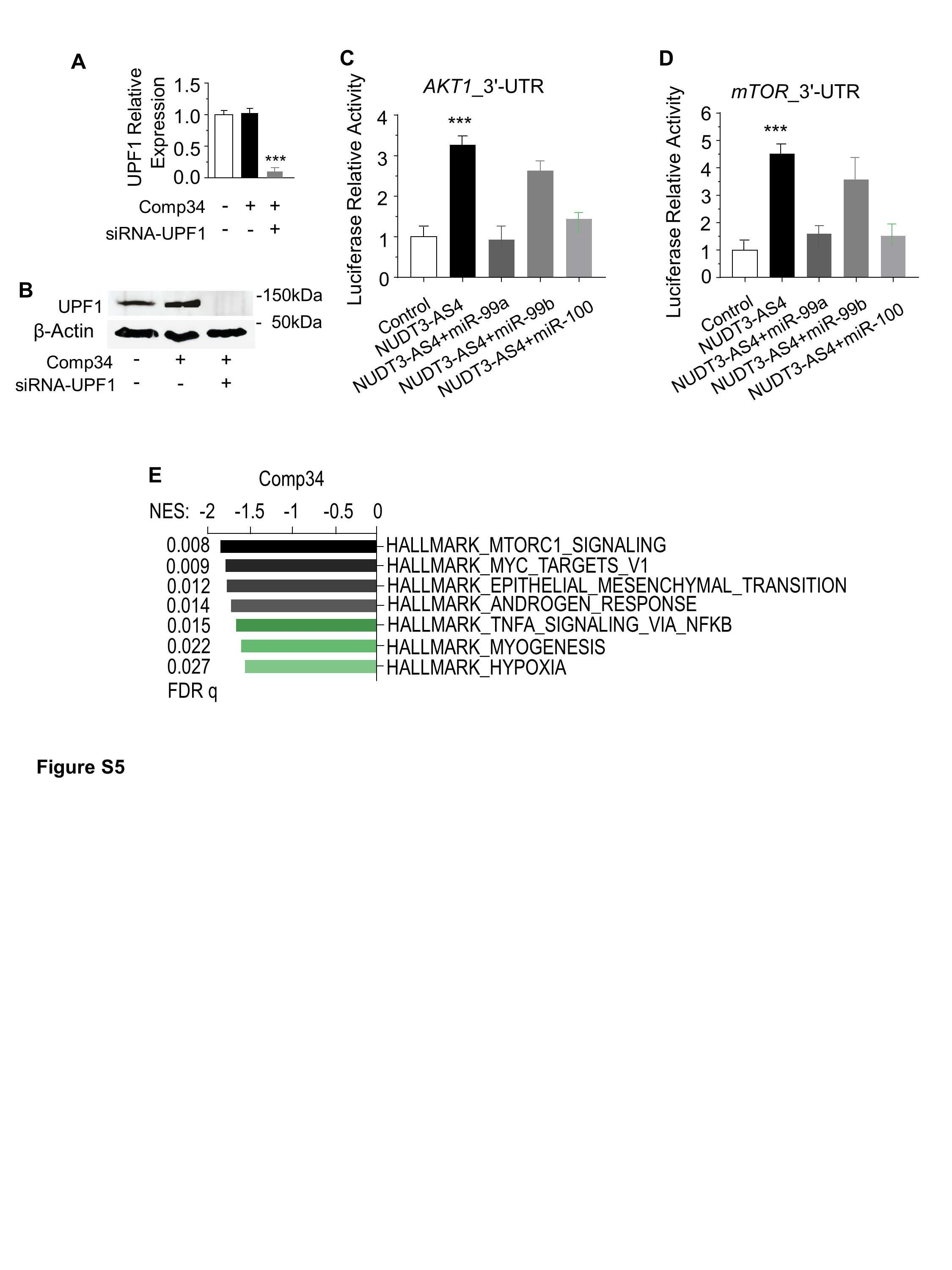

Supplement: Supplementary file 5 — Supplemental Figure 5 [file 41419_2020_3235_MOESM5_ESM.tif]

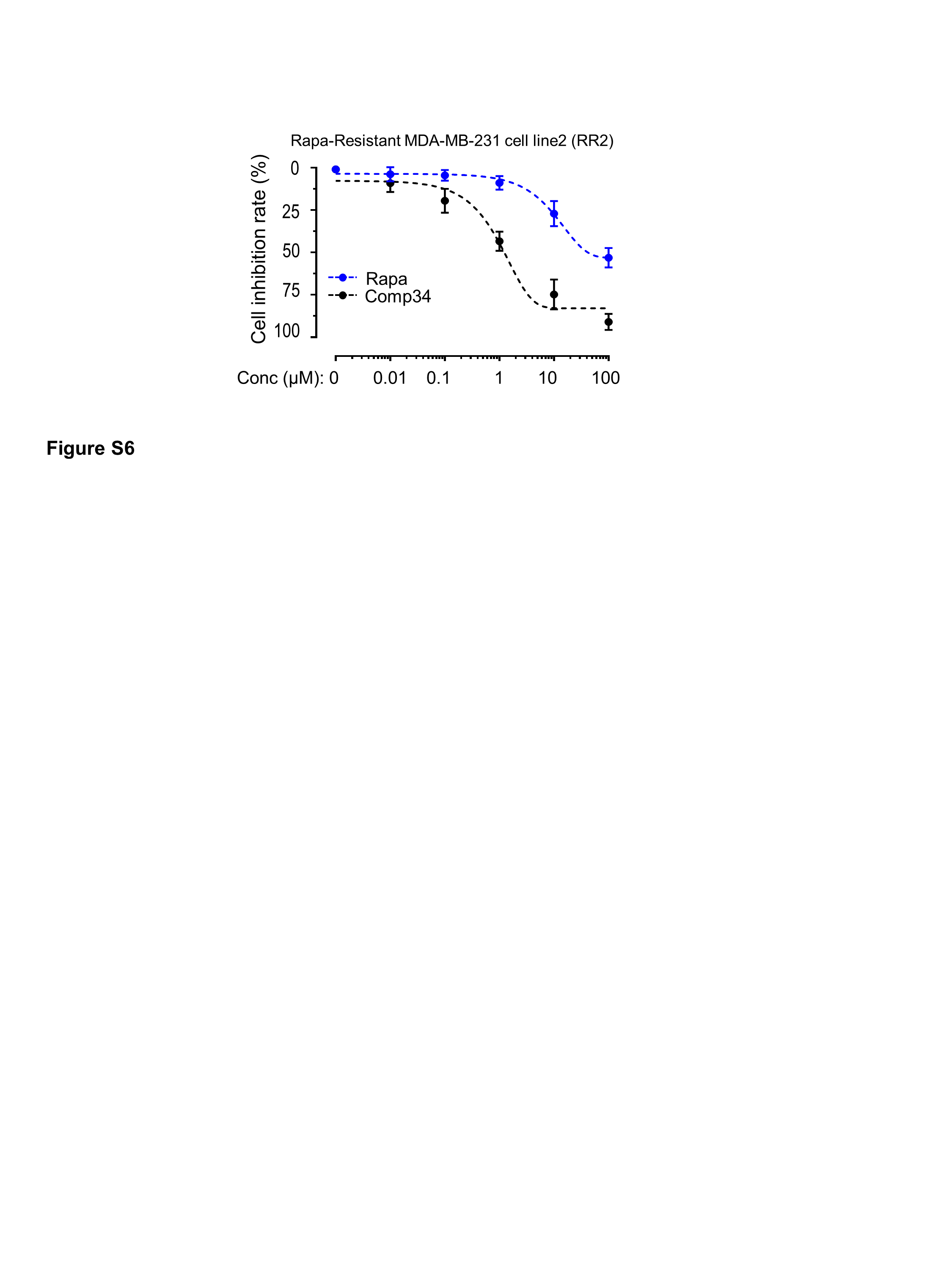

Supplement: Supplementary file 6 — Supplemental Figure 6 [file 41419_2020_3235_MOESM6_ESM.tif]

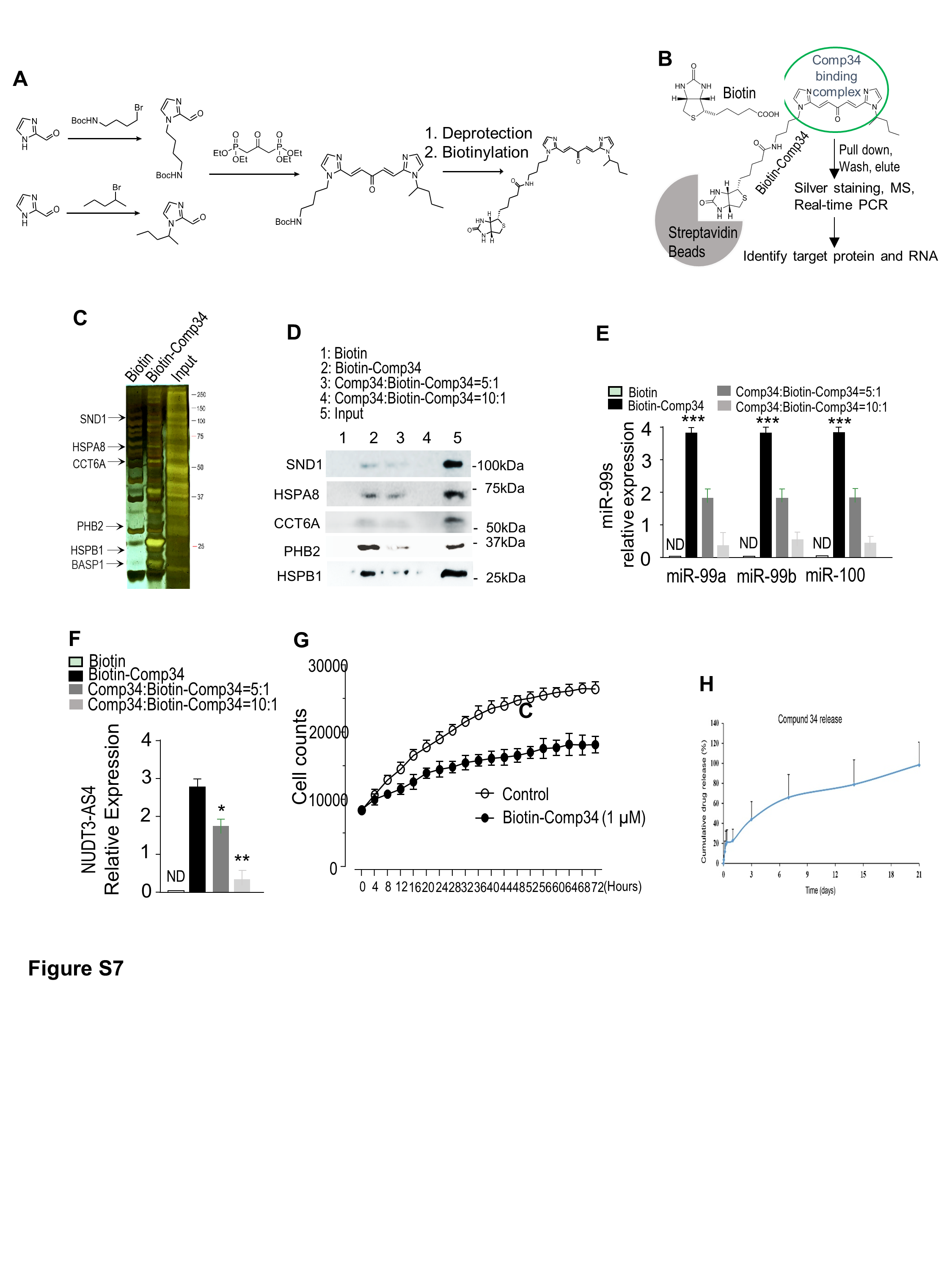

Supplement: Supplementary file 7 — Supplemental Figure 7 [file 41419_2020_3235_MOESM7_ESM.tif]
