# Supplementary material for: Comp34 displays potent preclinical antitumor efficacy in triple-negative breast cancer via inhibition of NUDT3-AS4, a novel oncogenic long noncoding RNA
Source: Cell Death Dis. 2020 Dec 11;11(12):1052. doi: 10.1038/s41419-020-03235-w (PMC7733521; doi:10.1038/s41419-020-03235-w)
Supplement: Supplementary file 8 — Supplemental tables [file 41419_2020_3235_MOESM8_ESM.docx]

Table S1 Binding sites between miR-99s and potential lncRNA.

| **miR-99a** |  | **miR-99b** |  | **miR-100** |  |
| --- | --- | --- | --- | --- | --- |
| **AL354740.1-204** |  | **AL354740.1-204** |  | **AL354740.1-204** |  |
| **Biotype:**  **retained_intron** | **Score:**  **0.801** | **Biotype:**  **retained_intron** | **Score: 0.814** | **Biotype:**  **retained_intron** | **Score: 0.799** |
| Binding area: 139-163; 7mer.  GA_ _ _ GG_ _ G_ _ _ UCAAGU_ _ _ _ _ _ _ _ _ A  _ _ UAU_ _ GG_ UGG_ _ _ _ _ _ UUUACGGGU_  _ _ . \| . _ _ \| . _ . \| \| _ _ _ _ _ _ \| . \| \| \| \| \| \| \| _  _ _ GUG_ _ C U_ GCC _ _ _ _ _ _ AGAUGCC CA_  _ _ _ _ _ UU_ _ A_ _ _ U_ _ _ _ _ _ _ _ _ _ _ _ _ _ A | | Binding area: 141-163; 7mer.  GAUA_ _ G_ GGU_ _ _ _ AA_ _ _ _ _ _ _ _ _ _ _ A  _ _ _ _ UG_ G_ _ _ GGUC_ _ GUUUUAC GGGU_  _ _ _ _ . \| _ . _ _ _ \| \| \| \| _ _ \| \| \| . \| \| \| \| \| \| \| _  _ _ _ _ GC _ U_ _ _ C CAG_ _ C AAGAUGCC CA_  _ _ _ _ _ _ G_ U_ _ _ _ _ _ C _ _ _ _ _ _ _ _ _ _ _ _ C | | Binding area: 139-163; 7mer.  GA_ _ _ GG_ GG_ _ _ UCAAGU_ _ _ _ _ _ _ _ _ A  _ _ UAU_ _ G_ _ UGG_ _ _ _ _ _ UUUAC GGGU_  _ _ . \| . _ _ \| _ _ . \| \| _ _ _ _ _ _ \| . \| \| \| \| \| \| \| _  _ _ GUG_ _ C _ _ GC C_ _ _ _ _ _ AGAUGCC CA_  _ _ _ _ _ UU_ AA_ _ _ U_ _ _ _ _ _ _ _ _ _ _ _ _ _ A | |
| Binding area: 3262-3282; 7mer.  UUCUUCA_ _ _ U_ _ _ _ U_ _ GUUAAC_ _ _ _ _ _ _  _ _ _ _ _ _ _ GC A_ _ _ UC _ GA_ _ _ _ _ _ ACGGGUU  _ _ _ _ _ _ _ . \| \| _ _ _ \| \| _ \| \| _ _ _ _ _ _ \| \| \| \| \| \| \|  _ _ _ _ _ _ _ UGU_ _ _ AG_ C U_ _ _ _ _ _ UGC CCAA  _ _ _ _ _ _ G_ _ _ UCU_ _ C_ _ AGA_ _ _ _ _ _ _ _ _ _ | | Binding area: 3262-3281; 6mer.  UUCUUCA_ _ _ UUC_ _ _ A_ _ _ AAC_ _ _ _ _ _ U  _ _ _ _ _ _ _ GCA_ _ _ _ UG_ GUU_ _ _ ACGGGU_  _ _ _ _ _ _ _ \| \| \| _ _ _ _ . \| _ \| \| \| _ _ _ \| \| \| \| \| \| _  _ _ _ _ _ _ _ C GU_ _ _ _ GC _ C AA_ _ _ UGC CC A_  _ _ _ _ _ _ G_ _ _ UC CA_ _ _ _ _ _ GA_ _ _ _ _ _ _ C | | Binding area: 3263-3282; 7mer.  UUCUUCAG_ _ _ UCU_ _ _ _ _ A AC_ _ _ _ _ _ _ _ _ _ _  _ _ _ _ _ _ _ _ CAU_ _ _ GAGUU_ _ _ _ _ _ _ ACGGGUU  _ _ _ _ _ _ _ _ \| \| . _ _ _ . \| \| \| \| _ _ _ _ _ _ _ \| \| \| \| \| \| \|  _ _ _ _ _ _ _ _ GUG_ _ _ UUCAA_ _ _ _ _ _ _ UGCCCAA  _ _ _ _ _ _ _ _ _ _ _ _ _ _ _ _ _ _ _ GCCUAGA | |
| **TTTY15** |  | **RP11-81F13.2** |  | **XLOC_013308** |  |
| **Biotype: lincRNA** | **Score: 0.793** | **Biotype:**  **antisense** | **Score: 0.781** | **Biotype:**  **lincRNA** | **Score: 0.798** |
| Binding area: 18-28; 6mer.  UUUUUUUUUUUUUUUUU_ _ _ AGC_ _ _ _ _ _  _ _ _ _ _ _ _ _ _ _ _ _ _ _ _ _ _ GUC _ _ _ CGGGUU  _ _ _ _ _ _ _ _ _ _ _ _ _ _ _ _ _ . \| \| _ _ _ \| \| \| \| \| \|  _ _ _ _ _ _ _ _ _ _ _ _ _ _ _ _ _ UAG_ _ _ GCC CAA  _ _ _ _ _ _ _ _ _ _ _ _ _ _ _ _ C_ _ _ AU_ _ _ _ _ _ _ | | Binding area: 416-441; 8mer.  U_ _ _ _ _ AAG_ _ ACUCUAC_ _ _ _ _ _ _ _ _ _ U  _ UGUAG_ _ _ UG_ _ _ _ _ _ _ UUCUAC GGGU_  _ . \| . \| . _ _ _ . \| _ _ _ _ _ _ _ \| \| \| \| \| \| \| \| \| \| _  _ GCGUU_ _ _ GC_ _ _ _ _ _ _ AAGAUGCC CA_  _ _ _ _ _ _ CC A_ _ C _ _ _ _ _ _ _ _ _ _ _ _ _ _ _ _ C | | Binding area: 54-65; 7mer.  UAAAGUCUUCCUUUC_ _ _ _ _ _ _ _ _ _ _ _ _ _ _ _ _ _ _ C  _ _ _ _ _ _ _ _ _ _ _ _ _ _ _ C AAGU_ _ _ _ _ _ UUAC GGGU_  _ _ _ _ _ _ _ _ _ _ _ _ _ _ _ \| \| \| \| \| _ _ _ _ _ _ . \| \| \| \| \| \| \| _  _ _ _ _ _ _ _ _ _ _ _ _ _ _ _ GUUCA_ _ _ _ _ _ GAUGCC CA_  _ _ _ _ _ _ _ _ _ _ _ _ _ _ U_ _ _ _ _ AGCCUA_ _ _ _ _ _ _ _ A | |
| Binding area: 42-55; 7mer.  UCAAUGCGCCAUA_ UG_ _ _ _ _ _ _ _ _ _ _ _ _ _ _ _ _ _ C  _ _ _ _ _ _ _ _ _ _ _ _ _ U_ _ AAGA_ _ GG_ _ _ _ AC GGGU_  _ _ _ _ _ _ _ _ _ _ _ _ _ . _ _ \| \| \| \| _ _ \| \| _ _ _ _ \| \| \| \| \| \| _  _ _ _ _ _ _ _ _ _ _ _ _ _ G_ _ UUCU_ _ C C_ _ _ _ UGCC C A_  _ _ _ _ _ _ _ _ _ _ _ _ _ _ UG_ _ _ _ AG_ _ UAGA_ _ _ _ _ _ A | |  |  | Binding area: 633-647; 7mer.  GCUUCUUUUUAUG_ UG_ _ A_ _ _ _ _ C_ _ _ _ _ _ _ _ _ _  _ _ _ _ _ _ _ _ _ _ _ _ _ U_ _ AA_ _ _ _ GG_ _ _ _ ACGGGUU  _ _ _ _ _ _ _ _ _ _ _ _ _ . _ _ \| \| _ _ _ _ \| \| _ _ _ _ \| \| \| \| \| \| \|  _ _ _ _ _ _ _ _ _ _ _ _ _ G_ _ UU_ _ _ _ CC_ _ _ _ UGCCCAA  _ _ _ _ _ _ _ _ _ _ _ _ _ _ UG_ _ CAAG_ _ UAGA_ _ _ _ | |
| **RP11-147L13.2** |  | **XLOC_013308** |  | **TTTY15** |  |
| **Biotype: antisense** | **Score:**  **0.789** | **Biotype: lincRNA** | **Score: 0.759** | **Biotype: lincRNA** | **Score: 0.795** |
| Binding area: 405-429; 8mer.  GCC _ _ _ C_ _ UUUUUUUUUUAA_ _ _ _ _ _ _ _  _ _ _ CGG_ UU_ _ _ _ _ _ _ _ _ _ _ _ UACGGGUU  _ _ _ \| \| \| _ \| . _ _ _ _ _ _ _ _ _ _ _ _ \| \| \| \| \| \| \| \|  _ _ _ GCC _ AG_ _ _ _ _ _ _ _ _ _ _ _ AUGCC CAA  _ _ A_ _ _ U_ _ _ _ _ _ _ _ _ _ _ _ _ _ _ _ _ _ _ _ _ _ | | Binding area: 54-65; 7mer.  UAAAGUCUUCCUUUC_ _ _ _ _ _ _ _ _ _ _ _ _ _ _ _ _ _ _ C  _ _ _ _ _ _ _ _ _ _ _ _ _ _ _ C AAG_ _ _ _ _ _ UUUAC GGGU_  _ _ _ _ _ _ _ _ _ _ _ _ _ _ _ \| \| \| \| _ _ _ _ _ _ \| . \| \| \| \| \| \| \| _  _ _ _ _ _ _ _ _ _ _ _ _ _ _ _ GUUC_ _ _ _ _ _ AGAUGCC CA_  _ _ _ _ _ _ _ _ _ _ _ _ _ _ C_ _ _ _ CAGCCA_ _ _ _ _ _ _ _ _ C | | Binding area: 18-28; 6mer.  UUUUUUUUUUUUUUUUU_ _ _ AGC_ _ _ _ _ _  _ _ _ _ _ _ _ _ _ _ _ _ _ _ _ _ _ GUC_ _ _ CGGGUU  _ _ _ _ _ _ _ _ _ _ _ _ _ _ _ _ _ . \| \| _ _ _ \| \| \| \| \| \|  _ _ _ _ _ _ _ _ _ _ _ _ _ _ _ _ _ UAG_ _ _ GCCCAA  _ _ _ _ _ _ _ _ _ _ _ _ _ _ _ _ C_ _ _ AU_ _ _ _ _ _ | |
| Binding area: 1075-1096; 6mer.  GUGGGA_ _ _ UCC_ _ _ _ CAGG_ C_ _ _ _ _ _ _  _ _ _ _ _ _ AC G_ _ _ UCGG_ _ _ _ C _ GCGGGUU  _ _ _ _ _ _ \| \| . _ _ _ \| \| \| \| _ _ _ _ \| _ . \| \| \| \| \| \|  _ _ _ _ _ _ UGU_ _ _ AGCC _ _ _ _ G_ UGCC CAA  _ _ _ _ _ G_ _ _ UCU_ _ _ _ UA_ _ _ A_ _ _ _ _ _ _ | | Binding area: 634-646; 6mer.  GCUUCUUUUUAUGU_ _ A_ _ _ _ C _ _ _ _ _ _ _ _ _ _ _ _ _ U  _ _ _ _ _ _ _ _ _ _ _ _ _ _ UG_ AAGG_ _ _ _ _ _ _ _ ACGGGU_  _ _ _ _ _ _ _ _ _ _ _ _ _ _ . \| _ \| \| \| \| _ _ _ _ _ _ _ _ \| \| \| \| \| \| _  _ _ _ _ _ _ _ _ _ _ _ _ _ _ GC _ UUC C_ _ _ _ _ _ _ _ UGC CC A_  _ _ _ _ _ _ _ _ _ _ _ _ _ _ _ _ G_ _ _ _ AGCCAAGA_ _ _ _ _ _ C | | Binding area: 42-55; 6mer.  UCAAUGCGCCAUA_ UG_ _ _ A_ _ _ _ _ _ _ _ _ _ _ _ _ _ C  _ _ _ _ _ _ _ _ _ _ _ _ _ U_ _ AAG_ _ _ GG_ _ _ _ AC GGGU_  _ _ _ _ _ _ _ _ _ _ _ _ _ . _ _ \| \| \| _ _ _ \| \| _ _ _ _ \| \| \| \| \| \| _  _ _ _ _ _ _ _ _ _ _ _ _ _ G_ _ UUC_ _ _ CC _ _ _ _ UGCC CA_  _ _ _ _ _ _ _ _ _ _ _ _ _ _ UG_ _ _ AAG_ _ UAGA_ _ _ _ _ _ A | |
| **XLOC_013308** |  | **RP11-379L18.1** |  | **RP11-147L13.2** |  |
| **Biotype: lincRNA** | **Score:**  **0.783** | **Biotype:antisense** | **Score: 0.744** | **Biotype:antisens**  **e** | **Score: 0.789** |
| Binding area: 54-62; 6mer.  UAAAGUCUUCCUUUCCAA_ _ _ _ _ _ _ _ _ _ C  _ _ _ _ _ _ _ _ _ _ _ _ _ _ _ _ _ _ GUUUACGGGU_  _ _ _ _ _ _ _ _ _ _ _ _ _ _ _ _ _ _ . \| . \| \| \| \| \| \| \| _  _ _ _ _ _ _ _ _ _ _ _ _ _ _ _ _ _ _ UAGAUGC CCA_  _ _ _ _ _ _ _ _ _ _ _ _ _ _ _ _ _ C _ _ _ _ _ _ _ _ _ _ A | | Binding area: 181-204; 7mer.  GGA_ _ AUC C_ U_ _ _ UAUU_ _ _ _ _ _ _ _ _ _ C  _ _ _ C G_ _ _ _ G_ UCG_ _ _ _ UUUUAC GGGU_  _ _ _ \| \| _ _ _ _ \| _ \| \| \| _ _ _ _ \| \| . \| \| \| \| \| \| \| _  _ _ _ GC_ _ _ _ C _ AGC _ _ _ _ AAGAUGCC CA_  _ _ _ _ _ GUU_ _ C_ _ _ C_ _ _ _ _ _ _ _ _ _ _ _ _ C | | Binding area: 405-429; 8mer.  GCC_ _ _ C_ _ UUUUUUUUUUAA_ _ _ _ _ _ _ _  _ _ _ C GG_ UU_ _ _ _ _ _ _ _ _ _ _ _ UAC GGGUU  _ _ _ \| \| \| _ \| . _ _ _ _ _ _ _ _ _ _ _ _ \| \| \| \| \| \| \| \|  _ _ _ GCC _ AG_ _ _ _ _ _ _ _ _ _ _ _ AUGCC CAA  _ _ A_ _ _ U_ _ _ _ _ _ _ _ _ _ _ _ _ _ _ _ _ _ _ _ _ _ | |
| Binding area: 633-645; 6mer.  GCUUCUUUUUAUGUU_ _ AA_ _ C_ _ _ _ _ _ _ _ _ _  _ _ _ _ _ _ _ _ _ _ _ _ _ _ _ GA_ _ GG_ _ _ _ AC GGGUU  _ _ _ _ _ _ _ _ _ _ _ _ _ _ _ \| \| _ _ \| \| _ _ _ _ \| \| \| \| \| \| \|  _ _ _ _ _ _ _ _ _ _ _ _ _ _ _ C U_ _ CC _ _ _ _ UGCC CAA  _ _ _ _ _ _ _ _ _ _ _ _ _ _ U_ _ AG_ _ UAGA_ _ _ _ _ _ _ | |  |  | Binding area: 1075-1096; 6mer.  GUGGGA_ _ _ UCC _ _ _ _ CAGG_ C _ _ _ _ _ _ _  _ _ _ _ _ _ AC G_ _ _ UC GG_ _ _ _ C_ GC GGGUU  _ _ _ _ _ _ \| \| . _ _ _ \| \| \| \| _ _ _ _ \| _ . \| \| \| \| \| \|  _ _ _ _ _ _ UGU_ _ _ AGCC _ _ _ _ G_ UGCC CAA  _ _ _ _ _ G_ _ _ UCA_ _ _ _ UA_ _ _ A_ _ _ _ _ _ _ | |
| **RP11-379L18.1** |  | **PROX1-AS1** |  | **RP11-81F13.2** |  |
| **Biotype:antisense** | **Score:**  **0.739** | **Biotype:antisense** | **Score: 0.714** | **Biotype:antisens**  **e** | **Score: 0.733** |
| Binding area: 180-204; 7mer. | | Binding area: 1068-1089; 7mer. | | Binding area: 418-442; 9mer. | |

| GG_ _ _ _ UC CGU_ _ _ UAUUU_ _ _ _ _ _ _ _ _ C  _ _ ACGA_ _ _ _ _ UC G_ _ _ _ _ UUUACGGGU_  _ _ \| \| . \| _ _ _ _ _ \| \| \| _ _ _ _ _ \| . \| \| \| \| \| \| \| _  _ _ UGUU_ _ _ _ _ AGC_ _ _ _ _ AGAUGCC CA_  _ G_ _ _ _ CU_ _ _ _ _ _ CU_ _ _ _ _ _ _ _ _ _ _ _ A | | CAUCA_ _ U_ _ _ _ _ _ _ GCUUUUG_ _ _ _ _ _ _ _ A  _ _ _ _ _ GC_ _ _ GUUGG_ _ _ _ _ _ _ UUAC GGGU_  _ _ _ _ _ \| \| _ _ _ \| \| . \| \| _ _ _ _ _ _ _ . \| \| \| \| \| \| \| _  _ _ _ _ _ C G_ _ _ CAGC C_ _ _ _ _ _ _ GAUGCC CA_  _ _ _ _ G_ _ UUC_ _ _ _ _ AA_ _ _ _ _ _ _ _ _ _ _ _ _ C | | UUG_ _ G_ _ _ _ GAC_ _ UAC U_ _ _ _ _ _ _ _ _ _  _ _ _ UA_ AAGU_ _ _ UC_ _ _ _ UCUAC GGGUU  _ _ _ . \| _ \| \| \| \| _ _ _ \| \| _ _ _ _ \| \| \| \| \| \| \| \| \| \|  _ _ _ GU_ UUCA_ _ _ AG_ _ _ _ AGAUGCC CAA  _ _ _ _ _ G_ _ _ _ _ _ _ _ _ C CU_ _ _ _ _ _ _ _ _ _ _ | |
| --- | --- | --- | --- | --- | --- |
| **RP11-81F13.2** | |  | | **PROX1-AS1** | |
| **Biotype:antisense** | **Score:**  **0.713** |  |  | **Biotype:antisens**  **e** | **Score: 0.704** |
| Binding area: 418-442; 9mer.  UUG_ _ G_ _ _ UGAC_ _ UAC U_ _ _ _ _ _ _ _ _ _  _ _ _ UA_ AAG_ _ _ _ UC_ _ _ _ UC UACGGGUU  _ _ _ . \| _ \| \| \| _ _ _ _ \| \| _ _ _ _ \| \| \| \| \| \| \| \| \| \|  _ _ _ GU_ UUC_ _ _ _ AG_ _ _ _ AGAUGCC CAA  _ _ _ _ _ G_ _ _ U_ _ _ _ _ C CU_ _ _ _ _ _ _ _ _ _ _ | |  | | Binding area: 1068-1091; 7mer.  CAU_ _ _ CUG_ _ _ _ _ CUUUUG_ _ _ _ _ _ _ _ A  _ _ _ C AG_ _ _ UUGGG_ _ _ _ _ _ UUAC GGGU_  _ _ _ \| \| . _ _ _ \| . \| \| . _ _ _ _ _ _ . \| \| \| \| \| \| \| _  _ _ _ GUU_ _ _ AGC CU_ _ _ _ _ _ GAUGCC CA_  _ _ U_ _ _ CA_ _ _ _ _ _ A_ _ _ _ _ _ _ _ _ _ _ _ _ A | |
| **PROX1-AS1** | |  | |  | |
| **Biotype:antisense** | **Score:**  **0.702** |  |  |  |  |
| Binding area: 1068-1094; 7mer.  _ _ _ C_ _ CU_ _ _ _ _ _ CUUUUG_ _ _ _ _ _ _ _ A  C AU_ AG_ _ GUUGGG_ _ _ _ _ _ UUACGGGU_  \| \| . _ \| \| _ _ . \| . \| \| . _ _ _ _ _ _ . \| \| \| \| \| \| \| _ GUG_ UC_ _ UAGCC U_ _ _ _ _ _ GAUGCC CA_  _ _ _ U_ _ _ _ _ _ _ _ _ _ A_ _ _ _ _ _ _ _ _ _ _ _ _ A | |  | |  | |

Table S2 Primers used for lentivirus production and shRNAs sequences

| Gene | | Sequence (5’→3’) |
| --- | --- | --- |
| AL354740.  1-204_sh1 | sense | CCGGGTATGAAGATGCTACTACTGGCTCGAGCCAGTAGTAGCATCTTCATACTTTTT |
|  | anti-sense | AATTAAAAAGTATGAAGATGCTACTACTGGCTCGAGCCAGTAGTAGCATCTTCATAC |
| AL354740.  1-204_sh2 | sense | CCGGGGTTATCAGCTAAAGACAAGACTCGAGTCTTGTCTTTAGCTGATAACCTTTTT |
|  | anti-sense | AATTAAAAAGGTTATCAGCTAAAGACAAGACTCGAGTCTTGTCTTTAGCTGATAACC |
| AL354740.  1-204_sh3 | sense | CCGGGACATTCTCAATAAAGACAGTCTCGAGACTGTCTTTATTGAGAATGTCTTTTT |
|  | anti-sense | AATTAAAAAGACATTCTCAATAAAGACAGTCTCGAGACTGTCTTTATTGAGAATGTC |
| shRNA- control | sense | GATCCGTTCTCCGAACGTGTCACGTTTCAAGAGAACGTGACACGT TCGGAGAACTTTTTTG |
|  | anti-sense | AATTCAAAAAAGTTCTCCGAACGTGTCACGTTCTCTTGAAACGTGA CACGTTCGGAGAACG |

Table S3 Primers used for quantitative real-time PCR and probe for RNA FISH

| Gene | | Sequence (5’→3’) |
| --- | --- | --- |
| Akt1 (ENST00000349310.7) | Forward | GCCTCTGCTTTGTCATGGAG |
|  | Reverse | AGCATGAGGTTCTCCAGCTT |
| mTOR (ENST00000361445.8) | Forward | GTGGTGGCAGATGTGCTTAG |
|  | Reverse | TTCAGAGCCACAAACAAGGC |
| PROX1-AS1 (ENST00000607258) | Forward | CTCGCCTTGGAGAGAGAGTT |
|  | Reverse | GCACAGTAGAACAGCCACAG |
| RP11-81F13.2 (ENST00000557642) | Forward | GCCACTGTTTGCCAGATACA |
|  | Reverse | ATCCAGGCTTCTCCCATCTG |
| RP11-379L18.1 (ENST00000587528) | Forward | GCCACTGTTTGCCAGATACA |
|  | Reverse | ATCCAGGCTTCTCCCATCTG |
| TTTY15 (ENST00000457658) | Forward | TTGCCGTTGGTGATTCAAGG |
|  | Reverse | CTGAACACAGGTCAGAAACCC |

| XLOC_013308 (line-  ZNF599-3) | Forward | TAAAGTCTTCCTTTCCAAGTTTACGGGTC |
| --- | --- | --- |
|  | Reverse | GCTTCTTTTTATGTTGAAAGGCACGGGTT |
| AL354740.1-204 (AL354740.1-204, ENST00000429998) | Forward | CTCCCACACCCTTTGATCCT |
|  | Reverse | AGTGTCCACCACTCCATCTG |
|  | Probe | AGAGAGCCCTCTCCATGCAGGG (5'Fam- 3'Tamra) |
