# Supplementary material for: Comp34 displays potent preclinical antitumor efficacy in triple-negative breast cancer via inhibition of NUDT3-AS4, a novel oncogenic long noncoding RNA
Source: Cell Death Dis. 2020 Dec 11;11(12):1052. doi: 10.1038/s41419-020-03235-w (PMC7733521; doi:10.1038/s41419-020-03235-w)
Supplement: Supplementary file 9 — Supplementary Figure Legends [file 41419_2020_3235_MOESM9_ESM.docx]

**Supplementary Figure Legends**

**Figure S1** (*Related to Figure 1*). **A.** Structures of a group of curcumin mimics. **B.** MDA-MB-231 cells were cultured with 100 nM Comp34 for 24 hours followed by incubation in the media without Comp34. stem cell population were analyzed by FACS using CD44 and CD24 antibodies. The results represent the mean percentage of stem cell population ± SD from 3 separate experiments. **P < 0.01 vs. Comp34. **C.** Western blotting (WB) analysis of the expression of AKT1, p-AKT, mTOR, p-p70S6K, p-S6, p-4EBP1 in the MDA-MB-436 cell line upon different concentrations of Comp34 treatment.

**Figure S2** (*Related to Figure 2*). **A**. MDA-MB-231 cells were transfected with miR-99s mimics. miR-99a, miR-99b and miR-100 expression levels were assessed by quantitative RT-PCR assays. miR-320 was used as internal control. n=3 in each group. ***P < 0.001 vs. control. **B**. miR-99s decrease the stemness of MDA-MB-231 cells. MDA-MB-231 cells were transfected with miR-99s mimics for 24 h, and stem cell populations were analyzed by FACS using CD44 and CD24 antibodies. The results represent the mean percentage of stem cell population ± SD, n=3. *P < 0.05 versus control. **C**. Top metabolic pathways from GSEA of downregulated or upregulated transcripts in miR-99a, miR-99b (**D**) and miR-100 (**E**) overexpression versus control using GSEA Hallmark MSigDB database.

**Figure S3** (*Related to Figure 3*). **A, B. The** expression correlation between of miR-99b and *AKT1, mTOR*. **C, D.** Overall survival in patients with breast cancer stratified according to AKT1 and mTOR expression status in their primary tumors.

**Figure S4** (*Related to Figure 4*). **A.** Quantitative real-time PCR analysis of expression level of 7 predicted lncRNAs binding with miRs-99s in MCF-12A and MDA-MB-231 cells. **P < 0.01, ***P < 0.001 vs. MCF-12A. **B.** NUDT3-AS4 was knocked down by NUDT3-AS4_sh lentivirus. MDA-MB-231 cells were retrovirally transduced with NUDT3-AS4_sh or control. Quantitative RT-PCR assays were performed to measure NUDT3-AS4 expression. **C**. miR-99s do not change the NUDT3-AS4 expression significantly in MDA-MB-231 cells determined by qPCR and RNA FISH assay (**D**). **E and F.** miR-99s does not change the luciferase activity of NUDT3-AS4 Mut1 or Mut2. Dual luciferase reporter assays were performed to test the binding between miR-99s and NUDT3-AS4 using the report gene constructs. Data are presented as relative luciferase activity of *Renilla* to Firefly luciferase activity. n = 6 independent experiments. **G**. NUDT3-AS4 overexpression does not change the miR-99s levels. MDA-MB-231 cells were cells were transfected with NUDT3-AS4 WT or the respective mutants. Quantitative RT-PCR assays were performed to measure the microRNA expression. **H**. Sequence alignment of miR-99s.

**Figure S5** (*Related to Figure 5*). siRNA targeting UPF1 significantly reduced mRNA (**A**) and protein expression (**B**) of UPF1 in MDA-MB-231 cells. **C.** NUDT3-AS4 increased the luciferase activity of *AKT1* and *mTOR* (**D**) 3’-UTR, and miR-99s abolished this increase. Dual luciferase reporter assays were performed to test the interactions of miR-99s and the targeting sequences in the *AKT1* and *mTOR* 3’-UTR using the reporter gene constructs. Data are presented as relative luciferase activity of *Renilla* to Firefly luciferase activity. n = 3 independent experiments. **P < 0.01, ***P < 0.001 vs. control. **E**. Top metabolic pathways from GSEA of downregulated transcripts in Comp34 treatment versus control using GSEA Hallmark MSigDB database.

**Figure S6** (*Related to Figure 6*). Rapamycin-resistant MDA-MB-231 cells (RR2) were treated with the indicated concentrations of Comp34 or rapamycin for 48 h. MTS cell proliferation assay was performed to determine the cell growth inhibition. Each dot and error bar on the curves represents mean ± SD (n = 6).

**Figure S7.** Identify the binding complex of Comp34. **A**. Strategy of synthesis of Biotin-Comp34. **B**. Strategy of Pull-down of Comp34-binding complex. **C**. The identified amino acids and peptides binding to Comp34. MDA-MB-231 cell lysates were incubated with Biotin-Comp34 and precipitated with strepavidin-agarose beads. The precipitated proteins were subjected to silver staining analysis. The arrowheads refer to the proteins that bind to Biotin-Comp34. **D**. The Comp34-binding proteins determined by MS were verified by immunoblotting against the indicated antibodies. **E** and **F**. Quantitative real-time analysis showed Biotin-Comp34 binding RNA could be competed away by Comp34. **G**. Biotin-Comp34 inhibits MDA-MB-231 cells proliferation. **H**. The *in vitro* time course of drug release kinetics of PLGA-NP.

**Table S1** Binding sites between miR-99s and potential IncRNA.

**Table S2** Primers used for lentivirus production and shRNAs sequences

**Table S3** Primers used for quantitative real-time PCR and probe for RNA FISH
